# Rapid Natural Killer Cell Gene Responses, Generated by TLR Ligand-Induced Trained Immunity, Provide Protection to Bacterial Infection in *rag1^−/−^* Mutant Zebrafish (*Danio rerio*)

**DOI:** 10.3390/ijms26030962

**Published:** 2025-01-23

**Authors:** Preeti J. Muire, Larry A. Hanson, Lora Petrie-Hanson

**Affiliations:** Department of Comparative Biomedical Sciences, College of Veterinary Medicine, Mississippi State University, 240 Wise Center Drive, Starkville, MS 39762, USA; pmuire@luc.edu (P.J.M.); lah3@msstate.edu (L.A.H.)

**Keywords:** trained immunity, beta glucan, TLR ligands, tissue-resident NK cell subsets, *Danio rerio*, vaccine adjuvants

## Abstract

T and B cell-deficient *rag1^−/−^* mutant zebrafish develop protective immunity mediated by trained immunity. In mammals, trained immune responses can be induced by Toll-like receptor (TLR) ligands. This study evaluated protective trained immunity in *rag1^−/−^* zebrafish through exposure to TLR ligands (beta glucan, R848, poly I:C), RE33^®^ (a live-attenuated *Edwardsiella ictaluri* vaccine), or combinations thereof, followed by wild-type *E. ictaluri* challenge one month later. Survival analyses revealed that all TLR ligands and vaccine treatments provided significantly higher protection than the control, with beta glucan inducing significantly greater protection than RE33^®^, while R848 and poly I:C were equivalent to the vaccine. Survivals for the treatments were beta glucan 70%, beta glucan + RE33^®^ 60%, R848 + RE33^®^ 54%, poly I:C + RE33^®^ 50%, R848 49%, poly I:C 32%, RE33^®^ 24%, and control 0%. Gene expression analysis of kidney and liver tissues post challenge revealed that beta glucan training elicited early and strong increased expressions of *nklb* (5536 fold @ 6 hpi), *nkld* (147 fold @ 12 hpi), and *ifng* (575 fold @ 12 hpi) in the kidney, and *ifng* (1369 fold @ 6 hpi), *nkla* (250 fold @ 6 hpi), *nklb* (734 fold @ 6 hpi), *nklc* (2135 fold @ 6 hpi) and *nkld* (589 fold @ 6 hpi) in the liver. Principal component analysis (PCA) revealed that early kidney gene expressions at 6–12 h post secondary infection (*nkla* @ 12 hpi, *nklb* @ 6 and 12 hpi, *nklc* @ 6 and 12 hpi, *nkld* @ 6 and 12 hpi, *ifng* @ 6 and 12 hpi, *t-bet* @ 6, 12 and 48 hpi, and *nitr9* @24 hpi) in the kidney and liver (*nkla*, *nklb*, *nklc*, *nkld*, *ifng*, *t-bet* and *nitr9* @ 6 hpi) were associated with the highest survival. This study highlights that TLR ligand-induced trained immunity boosts innate immunity and survival, with NK cell subpopulations in kidney and liver tissues responding differently to mediate protective responses.

## 1. Introduction

Trained immunity is a form of innate immune memory characterized by the enhanced ability of innate immune cells to respond more effectively to subsequent exposures to pathogens. Unlike classical innate immune responses, which are rapid but transient and non-specific, trained immunity involves long-lasting functional reprogramming of innate immune cells. This reprogramming enables these cells to respond more quickly with enhanced functions to future challenges, even those caused by unrelated pathogens. Unlike adaptive immunity, which relies on antigen-specific receptors and clonal expansion, trained immunity is mediated by epigenetic and metabolic changes that enhance innate immune readiness. Classical innate immune responses act as the first line of defense, using germ-line encoded pathogen recognition receptors (PRRs) to detect non-self pathogen-associated molecular patterns (PAMPS). These responses are immediate and essential for controlling infections, but do not have memory or sustained functional changes. Trained immune responses are ‘improved’ classical innate immune responses. They are distinguished by their ability to ‘remember’ previous encounters and maintain functional changes.

Several fish models have provided evidence for trained immunity, supporting the distinction between classical innate responses and the enhanced memory characteristics of trained immunity. Recombinase activating gene 1^−/−^ (*rag1^−/−^)* zebrafish, which lack functional adaptive immune cells due to the absence of V(D)J recombination [1], are an established model for studying trained immunity. Using adoptive cell transfers, it was demonstrated that innate immune cells of *rag1^−/−^* zebrafish mediated enhanced protective immunity for up to one month, in the absence of lymphocyte-based immunity [2]. Further studies suggested gene expressions underlying trained immunity in the MT zebrafish model [3,4]. The *rag1^−/−^* model also demonstrated long-term protection one month after gavage bacterial exposure [5]. Single-cell sequencing studies have revealed that specific innate immune cell types, including macrophages [6] and neutrophils [7] undergo trained immunity in zebrafish. These studies provide detailed transcriptional and epigenetic profiles, identifying key cell populations and molecular pathways, such as H3K4me3-driven reprogramming, that contribute to enhanced immune responses upon re-exposure to pathogens. These findings align with other observations in carp, where trained immune mechanisms were demonstrated in head-kidney-derived macrophages [8]. Similarly, epigenetic modifications to H3K4 and H3K27 in channel catfish anterior kidney leukocytes were associated with increased survival during *Edwardsiella ictaluri* and *E. piscicida* infections, improved phagocytic efficiency, and upregulated phagocytosis-related genes [9], demonstrating trained immunity. In this study, the enhancer modification H3K4me1 upregulated the regulation of actin cytoskeleton, mitogen-activated protein kinase (MAPK) signaling, endocytosis, focal adhesion, extracellular matrix receptor interaction and cell adhesion molecule pathways. The promoter modification H3K4me3 upregulated Toll-like receptor signaling, phagosome, MAPK signaling and advanced glycation end products–receptor for advanced glycation end products (AGE-RAGE) signaling pathways. The promoter modification H3K27ac upregulated Toll-like receptor signaling, retinoic acid-inducible gene-1 (RIG-1)-like receptor signaling, regulation of actin cytoskeleton and calcium signaling pathways, while the polychrome repressor modification H3K27me3 modification downregulated the Wnt signaling pathway [9]. There are practical benefits of trained immunity in fish. Because it is less specific, trained immunity can provide increased resistance to many pathogens. After one training event, fish can be protected against several diseases. Further, innate defenses are fully functional during early stages of fish development and at temperatures that may restrict acquired immunity.

In humans, trained immunity has the advantage of providing protection against diseases during stages or conditions when acquired immunity may be less functional. Trained immunity is possible through epigenetics, or changes caused by modifications of gene expression rather than alterations in the DNA sequence or genetic code itself. Trained immunity results from shifts in transcriptional regulation caused by epigenetic changes to the chromatin of innate immune cells [10] after PAMP exposure. Chromatin determines how the DNA is accessed and transcribed. Specific modifications of the histones influence the transcription of the genes in the region of the modified chromatin [11]. The genomic locations of the histone modifications are coordinated and cause broad changes in functional pathways of the innate immune cells and influence the type and magnitude of their responses when a pathogen is encountered. These changes result in stronger cellular defenses and increased survival. Trained immunity specifically relies on epigenetic changes to histones H3 and H4 [10].

In humans, multiple immune cells mediate trained immune responses, including monocytes/macrophages, natural killer (NK) cells, dendritic cells (DCs), and neutrophils [11,12]. Hematopoietic stem cells can also be trained and establish and maintain trained immunity [13,14]. In zebrafish tissues, leukocyte subsets are diverse and specialized. Hepatic macrophages have been demonstrated to undergo H3K4me3 modifications to enhance antimicrobial functions and repair liver injury during sepsis [15]. Macrophage and dendritic cell subsets have been identified in multiple zebrafish tissues and unique tissue-specific functions have been revealed [16]. Tissue-resident macrophages and neutrophils are involved in cardiac tissue repair and regeneration [17].

Trained immunity can be induced by multiple microbial components, including PAMPS and Toll-like receptor (TLR) ligands [18]. TLRs are pattern recognition receptors (PRRs) that, when activated, stimulate immune cells to release inflammatory molecules including cytokines, interleukins, and chemokines [19]. In aquaculture, beta glucan (TLR2/4 ligand) and poly I:C (TLR3 ligand) are well studied. The use of poly I:C to induce protection against fish viruses has been demonstrated [20,21]. (20, 21). Additionally, the upregulation of teleost TLR7 and 8, following viral infections suggests their antimicrobial role [22]. Beta glucan is an immune stimulant with long lived effects in aquaculture [8]. Beta glucan-induced trained immunity provided increased protection against bacterial diseases in MT zebrafish and channel catfish [5,9]. Resiquimod, or R848, is an immune modulator that binds to TLR7 and 8 and recognizes intracellular viral and bacterial RNAs localized in the endosome of infected cells [23]. R848 has been used as an immune modulator in zebrafish [5], flounder [24] and pompano [25]. RE33^®^ is a live-attenuated vaccine strain of *E. ictaluri* [26] and has been used in multiple studies with our zebrafish model [27].

NK cell lysins have been identified in various fish species, and NK lysin a, b, c and d have been described in zebrafish [28,29]. Tbox-21, or *t-bet*, is a T and NK cell transcription factor essential for the maturation of NK cells [30]. Nitr9 is an activating receptor characterized on NK cells in zebrafish [31,32]. Interferon gamma (*ifn*g) is produced by activated T and NK cells and the level of *ifn*g expression is correlated to cell cytotoxic activity [33].

The purpose of this study was to determine which of the TLR ligands induce the strongest trained immune response and mediate protection against live bacterial infection in MT zebrafish using ligands that stimulate different TLRs: beta glucan, a TLR 2/4 ligand, R848, a TLR7/8 ligand, and poly I:C, a TLR 3 ligand. To accomplish this, MT zebrafish were exposed to either beta glucan, R848, poly I:C, RE33^®^, beta glucan + RE33^®^, R848 + RE33^®^ or poly I:C + RE33^®^. After 1 month, fish were exposed to a LD80 of *E. ictaluri.* At 6, 12, 24 and 48 hours post exposure, kidney and liver tissues were removed and gene expression analyses performed. Survival was determined for each treatment. Principal component analysis (PCA) with survival as the outcome used survival and gene expressions associated with each treatment to determine which inter-correlated gene expression patterns increased survival of MT zebrafish after bacterial exposure. To gain another perspective on trained immunity and visualize cell phenotypes in beta glucan-, R848- and RE33^®^-exposed zebrafish, we examined cell expression of nitr9 and macrophage protein expressing gene-1 on kidney and liver leukocytes isolated at 24 and 48 hours post the training event, and 24 and 48 hours post the bacterial exposure after induction of trained immunity. Nitr9 and mpeg1 are genetic markers of NK cells and macrophages, respectively.

## 2. Results

### 2.1. Survival Trials

**Figure 1:**
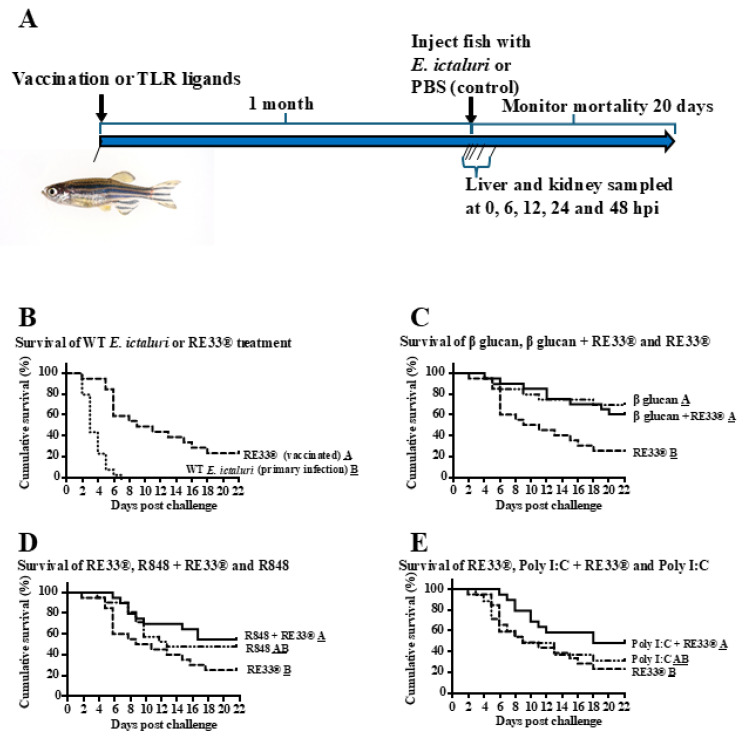
Workflow and survival analyses in *rag1^−/−^* mutant zebrafish treated with TLR ligands followed by bacterial challenge. (**A**). Workflow for zebrafish trained immunity study. Zebrafish were treated with TLR ligands followed by bacterial exposure to assess survival and gene expression. The data presented are from the experiment repeated three times. (**B**–**E**) Survival curves of *rag1^−/−^* mutant zebrafish treatment groups after challenge with WT *E. ictaluri* one month after the designated treatment. Curves that are significantly different have different underlined letter designation within the comparison (*p* < 0.05). The comparisons are (**B**) naïve fish (control) and RE33-vaccinated fish; (**C**) RE33^®^vaccinated fish, beta (β) glucan + RE33^®^-vaccinated fish or beta (β) glucan-treated fish; (**D**) RE-33^®^-vaccinated fish, R848 + RE33^®^-vaccinated fish and R848-treated fish; (**E**) RE33^®^-vaccinated fish, poly I:C + RE33^®^-vaccinated fish and poly I:C-treated fish.

RE33^®^-exposed MT zebrafish demonstrated significantly higher survival than non-exposed control fish when injected with a field isolate of WT *E. ictaluri* 4 weeks after innate training (Figure 1B, Table 1). Both beta glucan-induced training and beta glucan + RE33^®^ co-administration provided significantly greater survival than RE33^®^ (Figure 1C, Table 1). R848-induced training provided protection equivalent to RE33^®^. Trained immune protection induced by co-administration of R848 + RE33^®^ was equivalent to protection induced by R848 alone, and significantly greater than protection induced by RE33^®^ alone (Figure 1D, Table 1). Poly I:C-induced protection was not significantly greater than RE33^®^. However, protection induced by co-administration of poly I:C + RE33^®^ was significantly greater than RE33^®^ alone (Figure 1E, Table 1). Survival between the co-administered treatments beta glucan + RE33^®^ and R848 + RE33^®^ were not significantly different. However, both treatments demonstrated significantly higher survival than survival induced by exposure to poly I:C + RE33^®^. Moribund fish demonstrated clinical signs consistent with *E. ictaluri* and the progression of enteric septicemia of catfish (ESC) in zebrafish [27]. *E. ictaluri* was re-isolated from randomly sampled moribund fish. All control fish that were injected with WT *E. ictaluri* died by day 7 of the trial.

### 2.2. Quantitative Gene Expression

#### 2.2.1. Kidney

Kidney tissues from treated fish were differentially responsive to the bacterial challenge (Table 2, Supplementary Figures S1 and S2, Supplementary Table S1). Gene expression changes were negligible in the PBS injected fish. Naïve fish that were challenged showed moderate induction (1–100 fold) of *nitr9* at 6, 12 and 48 hpi, moderate induction of *nkla* at 48 hpi and high induction (101–1000 fold) of *ifn*g at 24 hpi. A strong response to infection was demonstrated by beta glucan-trained fish. Kidney tissues demonstrated a rapid response with very high induction (>1000 fold) of *nklb* at 6 hpi and high inductions of *nkld* and *ifn*g at 12 hpi. *Ifng* decreased to moderate expression at 24 hpi and *nkla* was moderately induced at 48 hpi. Kidney tissues from beta glucan + RE33^®^-trained fish demonstrated high induction of *nkld* at 12 and 24 hpi, and *ifn*g at 48 hpi and moderate induction of *nkla* at 48 hpi. Kidney tissues from RE33^®^-trained fish demonstrated very high induction of *t-bet* at 6 hpi and moderate induction of *nkla* at 48 hpi. R848-trained fish demonstrated a moderate induction of *nkla* at 48 hpi, while the R848 + RE33^®^-trained fish demonstrated high induction of *nkld* at 24 hpi and *ifn*g and moderate induction of *nkla* at 48 hpi.

#### 2.2.2. Liver

Liver tissues demonstrated a stronger responsive profile (Table 2, Supplementary Figures S3 and S4, Supplementary Table S1). The PBS injected fish showed mild responses. The naïve fish that were challenged showed very high expression of *ifn*g at 48 hpi and high expression at 6 hpi, Moderate expressions of *ifng* were documented at 12 hpi and *nklc* at 48 hpi. The beta glucan-trained fish had a more rapid response as was seen in the kidney with very high induction of *ifn*g and *nklc* at 6 hpi, and high induction of *nkla*, *nklb* and *nkld* at 6 hpi. Liver tissues from the beta glucan + RE33^®^-trained fish demonstrated the most induced genes, with very high induction of *ifn*g and *t-bet* at 24 hpi, and high induction of *nkld* at 12 hpi, and high induction of *nitr9*, *nkla*, *nklb*, *nklc* and *nkld* at 24 hpi. Liver tissues from the RE33^®^-trained fish demonstrated very high induction of *ifn*g at 48 hpi, high induction of *nitr9* at 6 hpi, and *nkla* and *nkld* at 12 hpi. Liver tissues from the R848-trained fish demonstrated high induction of *nitr9* at 12 hpi while the R484 + RE33^®^-trained fish demonstrated very high induction of *ifng* at 48 hpi and high induction of *nitr9* at 24 hpi. To gain the best understanding of the trained immunity-induced secondary gene responses associated with survival, we performed principal component analysis on the survival and gene expressions from the kidney and liver tissues of the beta glucan, R848, RE33^®^, beta glucan + RE33^®^ and R848 + RE33^®^-treated MT zebrafish.

### 2.3. Principal Component Analysis

Dealing with numerous gene expression fold changes and time points and associating these with survival can be challenging to interpret. Using the expression of multiple immune genes over time in principal component analysis (PCA) is a statistical approach to assess and compare the effectiveness and survival of different treatments. PCA used gene expression data (time and relative expression) and transformed it into principal components that were ordered by the degree to which they explain the survival data. PCA plotting demonstrated gene expression patterns. Treatments that led to similar responses were clustered together. This visualization helped identify which treatments and gene expressions had the most similar or different effects on survival. When survival was plotted within these groupings, the relationship of treatments and gene expressions to survival was demonstrated.

The PCA biplot analysis illustrated how survival (active observations) occurred relative to gene expressions and time (active variables) and suggests that different innate immune training treatments resulted in different patterns of NK cell gene expression. Each arrow on the biplot represents a gene at a specific time point, with the arrow’s length indicating how strong the gene/time point was in the overall gene expression pattern. F1 (the first principal component), represents the most dominant trend in gene expression changes. F2 is the second principal component and represents the second most dominant trend in gene expression that is independent of F1. Together, F1 and F2 demonstrate differences in gene expression and survival. The upper right quadrant in which F1 and F2 are both positive is where most of the gene expression variances are located, and these are the variables that had the greatest effect on survival. The treatment in this quadrant was correlated to strong immune gene activation. Gene expressions occurring in the upper left quadrant represent a different profile, and survival in this quadrant resulted from different gene expressions and pathways. Survival from treatments that occur in the lower quadrants was correlated with gene expressions or pathways that were different to in the upper quadrants, or from gene expressions that did not have as great an effect on survival.

**Figure 2:**
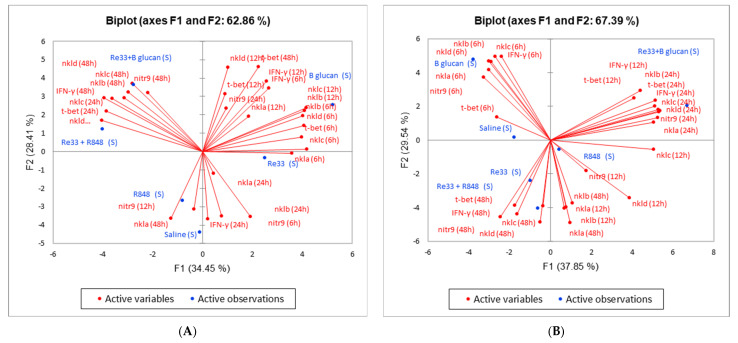
Principal component analysis (PCA) of vaccination treatments (active observations) associated with differential gene expressions (active variables) in (**A**). kidney marrow and (**B**) liver tissues. For each vaccination treatment, *ifng*, *t-bet*, *nitr9*, *nkla*, *nklb*, *nklc* and *nkld* were determined at 6, 12, 24 and 48 h post bacterial challenge. S refers to data collected after the secondary exposure (bacterial challenge) one month after induction of trained immunity. The vaccinations with the highest survival were beta glucan, beta glucan + RE33^®^ and R848 + RE33^®^. Red lines represent genes at specific time points, with the length indicating how strong the gene/timepoint was in the overall gene expression pattern.

The PCA F1 of vaccination treatments according to differential gene expressions in kidney tissue plotted six of the 6 hpi genes (*nkla*, *nklb*, *nklc*, *nkld*, *t-bet* and *ifn*g), and six of the 12 hpi genes (*nkla*, *nklb*, *nklc*, *nkld*, *t-bet* and *ifn*g) and *nitr9* and *t-bet* at 48 hpi in the upper right quadrant. The active observation beta glucan survival was in this quadrant, suggesting that it was strongly influenced by and associated with these gene expressions (Figure 2A and Supplementary Figure S5).

The PCA F2 of kidney included three of the 24 hpi genes (*nklc*, *nkld* and *t-bet*) and five of the 48 hpi genes (*nklb*, *nklc*, *nkld*, *ifn*g and *nitr9*) were in the upper left quadrant. The active observations beta glucan + RE33^®^ survival and R848 + RE33^®^ survival were in this quadrant, suggesting that they are strongly influenced by and associated with these gene expressions. The active observations R848 survival and RE33^®^ survival were in the lower quadrants, suggesting that these differ from the other treatments, and the immune responses induced by these treatments had weaker gene expression patterns or were more influenced by other genes (Figure 2A and Supplementary Figure S5).

The PCA F1 of vaccination treatments according to differential gene expressions in liver tissue included two of the 12 hpi genes (*t-bet* and *ifn*g) and all the 24 hpi genes (*nkla*, *nklb*, *nklc*, *nkld*, *t-bet*, *nitr9* and *ifn*g) in the upper right quadrant. The active observation beta glucan + RE33^®^ survival was in this quadrant, suggesting that it was strongly influenced by and associated with these gene expressions (Figure 2B and Supplementary Figure S6). All the six hpi genes (*nkla*, *nklb*, *nklc*, *nkld*, *ifn*g, *t-bet* and *nitr9*) were in the upper left quadrant (Figure 2B and Supplementary Figure S6). The active observation beta glucan survival was in this quadrant, suggesting that it was strongly associated with these gene expressions.

### 2.4. Expression of MPEG-1+ and NITR9+ Leukocytes in Kidney and Liver Tissues

#### 2.4.1. Kidney

**Figure 3:**
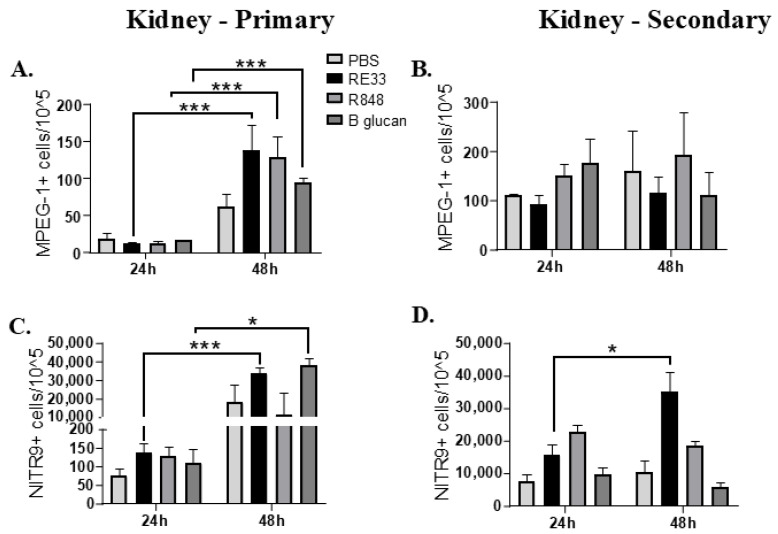
Flow cytometric analysis of *rag1^−/−^* mutant zebrafish MPEG-1+ kidney macrophages (**A**) 24 and 48 h post injection (hpi) with R848, beta glucan or RE33^®^ and (**B**) at 24 and 48 hpi of WT *E. ictaluri* one month after induction of trained immunity. (**C**) Flow cytometric analysis of *rag1^-/-^* mutant zebrafish NITR9^+^ kidney natural killer cells at 24 and 48 hours post injection (hpi) with R848, beta glucan or RE33^®^ and (**D**) at 24 and 48 hpi of WT *E. ictaluri* one month after induction of trained immunity. The bar graphs illustrate the mean cell counts with error bars indicating the standard error of the mean (SEM). Statistical significance was indicated as * *p* < 0.05 or *** *p* < 0.001 when comparing RE33^®^, R848 and beta glucan groups.

Flow cytometry demonstrated MPEG-1+ kidney leukocytes significantly increased between 24 and 48 h following initial exposures to RE33^®^, R848 and beta glucan IC injections (Figure 3A, Supplementary Figure S7, Supplementary Table S2). After one month, there were no significant changes in the kidney MPEG-1+ cells between 24 and 48 hpi after WT *E. ictaluri* challenge for any treatments (Figure 3B, Supplementary Figure S7, Supplementary Table S2). NITR9+ kidney leukocytes significantly increased between 24 and 48 h following initial exposures to RE33^®^ and beta glucan IC injection (Figure 3C, Supplementary Figure S7, Supplementary Table S2). After one month, RE33^®^-trained NITR9+ kidney leukocytes significantly increased between 24 and 48 hpi in WT *E. ictaluri*-exposed fish from these treatments (Figure 3D, Supplementary Figure S7, Supplementary Table S2).

#### 2.4.2. Liver

**Figure 4:**
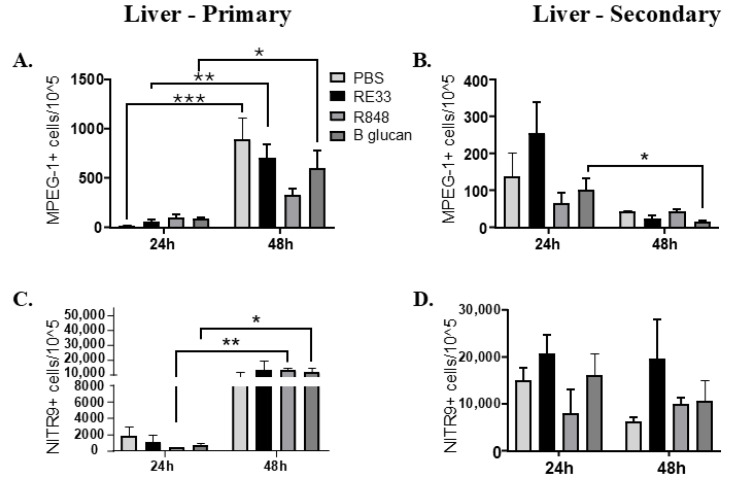
Flow cytometric analysis of *rag1^−/−^* mutant zebrafish MPEG-1+ liver macrophages (**A**) 24 and 48 h post injection (hpi) with R848, beta glucan or RE33^®^ and (**B**) at 24 and 48 hpi of WT *E. ictaluri* one month after induction of trained immunity. (**C**) Flow cytometric analysis of *rag1^-/-^* mutant zebrafish NITR9^+^ liver natural killer cells at 24 and 48 hours post injection (hpi) with R848, beta glucan or RE33^®^ and (**D**) at 24 and 48 hpi of WT *E. ictaluri* one month after induction of trained immunity. The bar graphs illustrate the mean cell counts with error bars indicating the standard error of the mean (SEM). Statistical significance was indicated as * *p* < 0.05, ** *p* < 0.01 or *** *p* < 0.001 when comparing RE33^®^, R848 and beta glucan groups.

MPEG-1+ liver leukocytes significantly increased between 24 and 48 h following initial exposures to RE33^®^ and beta glucan IC injections (Figure 4A, Supplementary Figure S8, Supplementary Table S2). After one month, MPEG-1+ liver leukocytes significantly decreased between 24 and 48 hpi after WT *E. ictaluri* challenge for the RE33^®^ and beta glucan training treatments (Figure 4B, Supplementary Figure S8, Supplementary Table S2). NITR9+ liver leukocytes significantly increased between 24 and 48 h following initial exposures to R848 and beta glucan (Figure 4C, Supplementary Figure S8, Supplementary Table S2). After one month, NITR9+ liver leukocytes did not significantly change between 24 and 48 hpi for any of the treatments (Figure 4D, Supplementary Figure S8, Supplementary Table S2).

## 3. Discussion

One of the hallmarks of trained immunity is protection against subsequent pathogen exposure. Our study demonstrates the induction of trained immunity with beta glucan, R848, poly I:C and RE33^®^ providing protection against the bacterial pathogen *E. ictaluri* 30 days after the training event in MT zebrafish. Beta glucan-induced trained immunity provided heterologous protection to IC administered *E. ictaluri* and *E. piscicida* in channel catfish [9] and gavage administered *E. ictaluri* in MT zebrafish [5]. An earlier study from our lab demonstrated adoptive cell transfers of innate immune cells from *E. ictaluri*-exposed MT zebrafish provided protection against *E. ictaluri* in naïve MT zebrafish [2].

Another hallmark of trained immunity is cellular metabolic changes characterized by pro-inflammatory genes being upregulated in a short time span after rechallenging with a secondary stimulus [34]. Enhanced cellular phagocytic functions were demonstrated after beta glucan-induced trained immunity in turbot [35], carp macrophages [8] and channel catfish [9]. Cellular metabolic changes result from histone methylation and acetylation reconfigurations. These changes were documented in channel catfish [9].

Trained immunity was demonstrated by transcriptomic studies using the MT zebrafish/*E. ictaluri* model when genes for cell receptor activation, signal transduction, cell proliferation and cytotoxic functions were upregulated [4] following secondary exposure to bacteria. These findings aligned with another study showing the correlation of upregulated inflammatory genes and gene pathways to beta glucan-induced trained immunity in catfish [9]. Many studies have documented the effects of TLR ligand exposures enhancing immune responses of fish (reviewed in [36,37]), but these studies documented immune stimulation or immune priming, not the long-term effects of trained immunity. Immune stimulation can last up to two weeks, and results in increased gene expression and cellular metabolic activity. Immune priming is a short-term heightened responsiveness that can be part of the adaptive immune response [11]. These early primary responses may be very different to the responses after epigenetic and metabolic reprogramming that is long term, and characteristic of trained immunity. Trained immunity involves a long-term functional reprogramming of innate immune cells that results in heightened responses upon secondary exposure, even after extended periods [11]. In our current study, the robust gene responses as early as 6 h after bacterial challenge in fish that received TLR ligand exposure one month earlier demonstrate a trained immunity effect, distinct from the transient immune activation seen during short-term immune stimulation or priming.

Principal component analysis of the kidney tissue and the separation of upregulated gene expressions into early (6 and 12 h) and late (24 and 48 h) responses suggest a timing difference in NK cell activation between beta glucan alone (upper right quadrant) and the combined beta glucan + RE33^®^ innate trainings (upper left quadrant). After beta glucan innate immune training, subsequent bacterial exposure resulted in a rapid initial response that resulted in early survival benefits and the highest survival of the innate trainings administered. Innate training with combined beta glucan + RE33^®^ resulted in a sustained immune response enhancing NK cell gene expressions at 24 and 48 h post bacterial exposure. These gene expressions resulted in survival that was equivalent to beta glucan alone but significantly greater than RE33^®^ alone. Beta glucan 6 and 12 hpi gene expressions in the upper right quadrant and the beta glucan + RE33^®^ 24 and 48 hpi gene expressions in the upper left quadrant suggest that different pathways may be involved in the trained immune responses of these two treatments. Active observations RE33^®^ and R848 survival were in the lower quadrants of the PCA. Although survival from each of these treatments was significantly higher than the control treatment, this suggests these treatments are less associated with the prominent genes or immune pathways associated with higher survival. Our findings suggest that the upregulation of transcription factor *t-bet* is very important for survival; the upregulation of this gene at 6, 12, and 48 h post bacterial exposure occurred in the upper right quadrant of the PCA biplot. *Nitr9* gene expression was the most diverse, with one of the four time points occurring in a different quadrant. This finding suggests that *nitr9* expression in the kidney is influenced by the expression of other genes or pathways and is not directly indicative of survival. In summary, beta glucan-induced trained immunity in the MT zebrafish kidney provides a quick NK cell response that results in the highest survival after secondary pathogen exposure. However, combining beta glucan with RE33^®^ innate training treatment results in equivalent protection and may create a more sustained trained immune response and prolonged activation, useful for extended pathogen resistance.

Principal component analysis in the liver revealed strong representation of beta glucan + RE33^®^ survival in the upper right quadrant and association with significant gene upregulation at 12 and 24 h post bacterial exposure. This suggests that in the liver, the beta glucan + RE33^®^ combination triggered a sustained immune response earlier compared to the kidney with the dominant factors affecting survival occurring mid-way through the sampling period. Early-to-mid-activation of liver NK cell-related genes induced by beta glucan + RE33^®^ innate training treatment were most associated with survival. In the upper left quadrant of the PCA biplot, beta glucan survival showed an association with early 6-h gene expressions, indicating a more immediate but possibly shorter-lived response in the liver. In our current study, PCA modeling links survival to temporal gene expression data. This statistical visualization approach is used in human disease research [38,39,40] but has not been used with fish disease research. PCA has been used to study zebrafish development [41] and the effects of environmental factors on fish [42] and oysters [43]. In our current study, PCA indicates the biological relevance of treatments that are more likely to have higher survival. The positioning of gene expressions in the upper quadrants suggests a stronger or more robust response associated with beta glucan and beta glucan + RE33^®^ survivals, suggesting that these treatments stimulate more immune pathways over time, resulting in a more comprehensive and prolonged immune response.

Interpreting the findings by tissue and time revealed that in the kidney, beta glucan had the greatest effect on gene expressions and initiated early protection, while beta glucan + RE33^®^ provided a more sustained activation, enhancing survival over a longer period. In the liver, beta glucan training induced early activation, but when combined with RE33^®^ shifted NK gene expressions to 12 and 24 h post exposure that resulted in a stronger response on the biplot. The complementary, or synergistic effect of beta glucan + RE33^®^ was very interesting. It appeared to induce a prolonged activation profile in both tissues, but on different timelines. In the kidney there was longer NK cell activation, and in the liver, there was an earlier sustained activation, possibly resulting in different immune benefits depending on the tissue. Varying immune cell populations in different tissues [3] can influence how each tissue responds to different TLR ligands and trained immunity inducers.

Our findings suggest that there are NK cell subpopulations in the kidney and the liver that may alter their function based on tissue microenvironment. Mammalian NK cells are composed of multiple subsets based on surface phenotype, immunological activity and anatomic distribution. Liver tissue harbors circulating NK cells and resident NK cells (restricted mobility in liver) [44]. Liver-resident NK cells exhibit anti-bacterial activity [45] and have different transcriptional profiles compared to circulating NK cells. MT zebrafish kidney and liver tissues have different NK-like cell profiles [3]. Similarly, zebrafish NK cells demonstrate cellular and functional diversification, including differential expression of NK-lysins, which are influenced by tissue-specific factors [28]. Mammalian tissue-resident memory NK cells have demonstrated specialized roles, determined by the tissue microenvironment and specific functional demands (reviewed in [46]. In our experiments, the liver and kidney show distinct timing and intensity in NK cell gene responses to immune stimuli. The differences in timing and survival associations between beta glucan, beta glucan + RE33^®^, and TLR training suggest that certain NK subpopulations in each tissue have specific roles and timing in immune responses to different ligands and support survival differently. Beta glucan training induces a response that is rapid in the kidney NK cell subpopulation, and longer to upregulate gene expression in the liver and supports higher survival. However, the combination of beta glucan + RE33^®^ induces a more rapid response in the liver and is associated with lower survival than beta glucan alone. An earlier response may correlate to a NK cell subpopulation that is more responsive to a ligand, or more abundant in a tissue. A longer response may suggest a NK cell subpopulation that may have a regulatory or memory function. We believe that differences in timing and intensity of gene expression responses between kidney and liver tissues suggest functionally diverse NK cell subpopulations as described by Pereiro [28]. Further studies of isolated NK cell subpopulations are needed to validate these interpretations.

Our flow cytometry data determined the levels of NK cell and macrophage biomarkers Nitr9 and MPEG-1, respectively, at 24 and 48 hpi after WT *E. ictaluri* challenge and found minimal changes in the cell populations of the trained fish after challenge. These findings suggest the upregulation of NK-associated genes at 24 and 48 h were not related to changes in Nitr9+ NK cell numbers and suggests NK cell gene expressions (6 and 12 hpi in the kidney and 12 to 24 hpi in the liver) were indicative of enhanced immune readiness not increased cell numbers.

In kidney tissue, increased numbers of Nitr9 and MPEG-1-positive cells in the primary response suggested that NK cells and macrophages responded to the initial training induced by beta glucan, RE33^®^ and R848. The lack of increased MPEG-1-positive cells following bacterial exposure after one month suggests that kidney trained immunity functional changes do not require increased MPEG-1-positive cell numbers. It is interesting to note that RE33^®^-induced training resulted in increased Nitr9+ cell numbers at 48 hpi following bacterial exposure after one month. In the PCA, kidney *nitr9* at 48 hpi was in the upper left quadrant and was associated with RE33^®^+beta glucan and RE33^®^+R848 survival and could result from memory NK cells, but this change in the population was not seen with the other treatments. 

In liver tissue, the increased MPEG-1-positive cells suggest that macrophages responded to RE33^®^ and beta glucan exposure. However, decreased MPEG-1-positive cells following bacterial exposure after one month suggest that RE33^®^ and beta glucan-trained liver macrophages may exhibit a regulated or restrained response after a secondary bacterial challenge, possibly to control inflammation. RE33^®^-, R848- and beta glucan-induced training resulted in significantly increased Nitr9+ cell numbers in the liver. However, none of the trained fish demonstrated a change in Nitr9+ numbers after a secondary bacterial challenge. In the PCA, *nitr9* at 48 hpi was in the lower left quadrant. This is very different to in the kidney tissue. A possible explanation is that the liver is involved in maintaining metabolic and immune balance, so prolonged or sustained immune activation is regulated to prevent inflammation. Temporally, the kidney and liver flow cytometry findings occurred after the initial gene responses correlated to increased survival. At 48 h post the secondary challenge, regulatory or suppressive immune responses would be required to control the heightened early responses associated with trained immunity.

This study demonstrated that TLR ligands induced trained immunity and protection to subsequent bacterial challenge in lymphocyte-deficient zebrafish, with the best protection provided by beta glucan. Incorporating these ligands into practical aquaculture practices can lead to increased production yields and disease resistance and reduce antibiotic use. Co-administration of TLR ligands with a live-attenuated vaccine increased survival above that of the vaccine alone, demonstrating that these agents can act as powerful adjuvants and improve vaccine efficacy. The basis of protection was rapid NK cell immune gene responses that varied between kidney and liver tissues, suggesting that tissue-resident NK cell subpopulations were responding differently to immune training induced by different ligands. These findings may translate to other commercially important fish species.

Future investigations should focus on uncovering the molecular mechanisms underlying trained immunity in NK cells and other innate immune cells across fish species. For aquaculture applications, research should prioritize exploring trained immunity induced by alternative ligands, identifying optimal adjuvants, and evaluating the long-term effects and sustainability of trained immune responses.

## 4. Material and Methods

### 4.1. Zebrafish Care

Zebrafish were raised and maintained at 28 °C in the specific-pathogen-free (SPF) hatchery in the College of Veterinary Medicine (CVM), Mississippi State University (MSU) following standard lab protocols. Experimental protocols were approved by the MSU Institutional Animal Care and Use Committee. A homozygous breeding colony of *rag1^−/−^* mutant (MT) zebrafish was previously established [1] and bred at the CVM-SPF hatchery and the experimental fish were progeny from that colony.

### 4.2. Innate Immune Training

A flow diagram representing the experiments performed is presented in Figure 1A. MT zebrafish were administered either a TLR ligand, attenuated bacteria, or co-administered a TLR ligand and attenuated bacteria. To accomplish this, MT zebrafish were intracoelomically (IC) injected with either beta glucan 50 μg/0.5 g of fish, poly I:C 50 μg/0.5 g of fish, R848 0.08 μL/0.5 g of fish, 1 × 10^4^ CFU live-attenuated vaccine strain of *Edwardsiella ictaluri* (RE33^®^) (26), beta glucan+RE33^®^, poly I:C+ RE33^®^ or R848+RE33^®^. Each of these compounds was dissolved in 10 μL of PBS and were injected using a 0.5 mL tuberculin syringe with a 29 G needle. Control fish were IC injected with 10 μL/fish of endotoxin-free PBS. Two tanks with n = 10 per tank were used per treatment and the entire experiment was repeated three times (total n = 60 per treatment).

### 4.3. Survival Trials

#### 4.3.1. Preparation of Bacterial Cultures

*E. ictaluri* (field strain 93146) identification was confirmed by biochemical analysis using the bioMerieux api20 strip (Bio Merieux, 69280 Marcy l’Etoile, France). Aliquots (0.5 mL) were stored in 20% glycerol at −80 °C until needed for trials, at which time one aliquot was thawed and added into Brain Heart Infusion broth and incubated in a shaker incubator at 30 °C overnight. Logarithmic phase cultures were obtained by dilution of the overnight culture 1:10 and grown until the optical density was 0.4 at 540 nm which corresponds to 10^8^ colony forming units (CFU) per mL. Culture purities were assessed and bacterial concentrations determined by plating serial dilutions on 5% sheep blood agar plates and observing the plates twice a day for 4 days.

#### 4.3.2. Lethal Dose Determination

In separate trials, MT mutant zebrafish were injected with *E. ictaluri*, (10^6^, 10^5^, 10^4^, 10^3^, 10^2^, or 10^1^ CFU/fish) to select the LD80 dosage for the protection exposures (to determine if the treatment exposures provided protection). Injections of MT zebrafish were performed using four replicate tanks per treatment with 15 fish per replicate. Additionally, 15 control fish were sham injected. Mortalities were recorded for 18 days post injection (dpi), and the dose at which 80% of the fish died was used for the protection exposure; this dose was 1 × 10^4^ CFU/fish.

#### 4.3.3. Bacterial Infections

Four weeks after innate immune training, the zebrafish were challenged by 1 × 10^4^ CFU/fish intracoelomic (IC) injections of virulent WT *E. ictaluri* (field strain 93146). Fish were observed and deaths recorded for 20 days. Fish were observed three times per day and moribund fish were euthanized by immersion in 340 ppm Tricaine methane sulfonate and then placed in a freezer. Randomly sampled fish were necropsied to determine the presence or absence of *E. ictaluri*.

### 4.4. Quantifying Gene Expression

For this section, we focused on the treatments that resulted in substantial survival. Innate immune training treatments were administered as described above with the omission of poly I:C and poly I:C +RE33^®^. Four weeks later, a bacteria challenge was performed as described above. In fish, kidney tissue is a hematopoietic tissue, equivalent to the mammalian bone marrow and is a primary lymphoid organ. Following the bacteria challenge, fish were euthanized in buffered 0.02% MS222 and liver and kidney tissues from fish (n = 5) were surgically removed at 6, 12, 24 and 48 h post bacterial injection (hpi) for each treatment. For basal expression, 0 h non-injected fish were sampled. Tissues were immediately transferred to 400 μL Trizol reagent (Zymo Research, Irvine, CA, USA) and homogenized following standard procedures in our lab [47]. Total RNA was extracted from each liver and kidney sample using RNA extraction kits (Zymo Research, Irvine, CA, USA) according to the manufacturer’s protocol. The quantity and purity of extracted total RNA was determined by NanoDrop ND-1000 and ND-8000 8-Sample Spectrophotometer and stored at −80 °C until used. 100 ng cDNA was prepared from RNA by using Super script III VILOTM cDNA Synthesis Kit (Invitrogen, Carlsbad, CA).

*Arp*, *ifnγ*, *t-bet*, *nitr9*, *nkla*, *nklb*, *nklc* and *nkld* gene expression was measured using real time quantitative PCR. The *t-bet* primer and probe (Table 3) were designed by Beacon Designer 8.0 software (BioRad) and Primer3 plus version 2.6 (GraphPad) software, respectively. The source of the other primers and probes are included in Table 3. All primers and probes were purchased from Eurofins MWG, Operon, Huntsville, AL, USA. Amplification of the ubiquitously expressed acidic ribosomal phosphoprotein (*arp*) gene was used for the internal control to normalize the gene expression data and validate extracted RNA integrity [48]. The amplification was performed in a 25 μL volume containing 10 μL target cDNA and 15 μL master mix containing: 8.8 μL nuclease-free water (GIBCO, Ultra PureTM), 1.5 μL MgCl2 (5 mM), 2.5 μL 10 × buffer, 0.5 μL dNTPs, 0.2 μL Taq Polymerase HS enzyme (Hot Start PCR Kit, TAKARA, Japan), 0.5 μL forward primer (20 μM), 0.5 μL reverse primer (20 μM) and 0.5 μL probe (10μM). Thermal cycler parameters for the PCR program were set as follows: 50 °C for 2 min, 95 °C for 10 min, 45 cycles of 95 °C for 15 s and 61 °C for 1 min. All samples (biological reps) were run in triplicates, i.e., 3 technical reps/sample. Gene expression levels of *ifnγ*, *t-bet*, *nitr9*, *nkla*, *nklb*, *nklc* and *nkld* were normalized with housekeeping gene, *arp*, expression levels. No significant differences were observed in the gene expressions between tissues prior to injecting *rag1^−/−^* zebrafish with TLR ligands or RE33^®^ and combinations described in Section 4.2.

### 4.5. Principal Component Analysis

To identify the genes most influential on survival, we used XLSTAT 2020.3.1.1000 to perform principal component analysis (PCA), a technique that transforms complex data into a set of principal components to simplify and interpret patterns in the data. Kidney and liver expressions of *ifng*, *t-bet*, *nitr9*, *nkla*, *nklb*, *nklc* and *nkld* at 6, 12, 24 and 48 h post bacterial exposure were the active variables and cumulative survival for each TLR ligand treatment were the active observations. PCA calculated Pearson’s correlation coefficients to construct a correlation matrix that examined all the gene expressions at different time points and identified how related they were. Variables that were highly correlated contributed similarly to a principal component, while less correlated variables contributed differently. Eigenvectors were derived from the correlation matrix and were used to transform the active variables into principal component 1 and principal component 2. The PCA quadrant biplot demonstrates the trends in gene expression associated with survival of different treatments. Each arrow on the biplot represents a gene at a specific time point and where the arrow is placed in the quadrant demonstrates how strongly it influenced the gene expression pattern. The location of the survival observation demonstrates how much that outcome was influenced by the gene expression pattern in a specific quadrant.

### 4.6. Nitr9 and Mpeg-1 Expression by Fluorescence-Activated Cell Sorting (FACS)

MT adult zebrafish (n = 15/group) were IC injected with either beta glucan 50 μg/0.5 g of fish, R848 0.08 μL/0.5 g of fish, 1 × 10^4^ CFU live-attenuated vaccine strain of *E. ictaluri* (RE33^®^) [26] or endotoxin-free PBS 10 μL/fish. After 24 or 48 h, fish were euthanized in buffered 0.02% MS222 and liver and kidney tissues surgically removed. Tissues from five fish were pooled for each biological replicate and three replicates were used per treatment. Tissues were mechanically dislodged with a Teflon pestle and passed through a 40 μm sterile filter. Cells were washed in Hank’s Balanced Salt Solution (HBSS) buffer without Ca^2+^ and Mg^2+^ at 500× *g* for 20 min. Leukocytes were enriched by separation on Histopaque^®^-1077 gradient (Sigma-Aldrich). Briefly, the cell suspension was layered on Histopaque^®^-1077 gradient at a ratio of 1:1 and centrifuged at 400× *g* for 30 min and the leukocyte layer was collected. Cells were labelled with a zombie green viability dye (BioLegend), incubated for 30 min at room temperature, and washed. This was followed by addition of purified rat anti-mouse CD16/CD32/mouse BD Fc block (BDPharmingen^TM^) (1:100) and staining with anti-zebrafish primary antibodies: anti-NITR9^90.10.5^ (1:20) (University of North Carolina at Chapel Hill Core Facility; https://www.med.unc.edu) and anti-MPEG-1 (1:40) (Anaspec^TM^) to the respective tubes. Before staining cells with anti-Nitr9 antibody, the cells were permeabilized with perm buffer (BDbiosciences^TM^) and Fc blocked to facilitate intracellular staining. Samples were incubated on ice for 45 min, washed and stained with secondary antibody goat anti-mouse IgG-PE for anti-Nitr9^90.10.5^ antibody and goat anti-rabbit IgG-APC for anti-MPEG-1 antibody followed by 15 min of incubation on ice. Cells were then washed in FACS (HBSS + 2% BSA) buffer and analyzed on a FACS Novocyte. Samples collected at this point are referred to as ‘trained’. Isotype controls were used as a negative control to help differentiate non-specific background signal from specific antibody signal. Rat IgG2b isotype control (Invitrogen, 02-9288), mouse IgG2b isotype control (Invitrogen, 02-6300) and rabbit IgG polyclonal isotype control (Abcam, ab37415) were used as negative controls as appropriate. The isotype controls for each fluor were stained using the isotype control as the primary antibody for 1 h followed by incubation with specific fluor for 30 min. Samples were incubated on ice until analyzed.

Flow cytometry analyses of kidney leukocytes involved forward scatter (FSC) and side scatter (SSC) determinations on a Calibur FACS. FSC represents cell size in diameter and SSC represents cell granularity, or complexity. Twenty thousand cells were collected from each sample. Cells were gated in three areas based on cell sizes and granularity. Unstained, fluorescence minus 1 (FMO), and isotype controls were used to set gates and determine positivity. The positive cells were calculated using the percentage of positive cells minus the number of positive ells for the isotype control. Results are presented as the mean number of cells positive for a specific antibody. Novoexpress software version 1.5 was used for analysis.

Four weeks after the training event, the fish were challenged with 1 × 10^4^ CFU/fish IC injections of a virulent WT *E. ictaluri* (strain #93146). A control group of naïve fish (n = 15) were injected with virulent WT *E. ictaluri*. Fish were euthanized in buffered 0.02% MS222. Liver and kidney tissues from fish were surgically removed at 24 and 48 h as described above. Leukocytes were collected and labeled with anti-Nitr9^90.10.5^ and anti-MPEG-1 following the same procedures described above.

### 4.7. Data Analysis and Statistical Evaluation

Survival curve analysis was performed by the Kaplan–Meier survival plot. The non-parametric statistic tests Gehan–Breslow–Wilcoxon test and Log ranked (Mantel–Cox) test were used to estimate the statistical significance between the survival curves of RE33^®^ only, TLR ligand groups only and RE33^®^ plus TLR ligand groups. The Gehan–Breslow–Wilcoxon method gives more weight to mortalities at early time points. The Log-rank (Mantel–Cox) test gives equal weight to all time points and is preferred. Relative gene expression was determined using the Pfaffl method [50]. Data obtained from qRT-PCR were expressed as fold change and were converted to log2 values. Data were analyzed by two-way ANOVA followed by Dunnet’s multiple comparison test. Data obtained from FACS were analyzed by two-way ANOVA followed by Tukey’s multiple comparisons test. Survival curve, qPCR and FACS data statistical analyses were performed using “GraphPad Prism version 6.00 for Windows, GraphPad Software, La Jolla, CA, USA, www.graphpad.com”. An alpha level of 0.05 was used to determine the significance of all analyses. A matrix of Pearson’s correlations demonstrated the gene expressions that were significantly correlated for each treatment. Principle component analysis XLSTAT 2020.3.1.1000 was performed using Pearson correlations, and examined survival was described by time-correlated gene expression patterns.

## Figures and Tables

**Table 1:** Statistical analyses of survival analyses of fish administered TLR ligands or RE33^®^ alone, or combinations of TLR ligands and RE33^®^. *Rag1^−/−^* mutant zebrafish were challenged with wild-type *E. ictaluri* four weeks following TLR ligands and/or RE33^®^ administration. Asterix * indicates statistical significance at *p* < 0.05.

Treatment	Breslow–Wilcoxon	Mantel–Cox	Hazard Ratio	95% CI of the Ratio
E33^®^ v control	*p* < 0.0001 *	*p* <0.0001 *	0.25	0.116 to 0.5364
Beta glucan v RE33^®^	*p* = 0.025 *	*p* = 0.014 *	0.34	0.143 to 0.7963
Beta glucan + RE33^®^ v RE33^®^	*p* = 0.001 *	*p* = 0.012 *	0.36	0.157 to 0.307
Beta glucan + RE33^®^ v beta glucan	*p* = 0.074	*p* = 0.626	0.77	0.2705 to 2.199
Poly I:C v RE33^®^	*p* = 0.354	*p* = 0.251	0.65	0.3005 to 1.402
Poly I:C + RE33^®^ v RE33^®^	*p* = 0.033 *	*p* = 0.050 *	0.48	0.2157 to 1.057
Poly I:C + RE33^®^ v poly I:C	*p* = 0.084	*p* =0.158	1.78	0.7626 to 4.195
R848 v RE33^®^	*p* = 0.174	*p* = 0.187	0.60	0.2802 to 1.313
R848 + RE33^®^ v RE33^®^	*p* = 0.019 *	*p* = 0.028 *	0.42	0.1870 to 0.950
R848 + RE33^®^ v R848	*p* = 0.452	*p* = 0.473	1.36	0.5678 to 3.286

**Table 2:** Summary of significantly differentially expressed genes in kidney or liver tissues of *rag1^−/−^* mutant fish at different hours post injection (hpi) with wild-type *E. ictaluri* four weeks following induction of trained immunity by Toll-like receptor (TLR) ligands or RE33^®^ alone, or combinations of TLR ligands and RE33^®^. Non-significantly expressed genes are not shown in this table. The genes reported are interferon gamma (*ifnγ),* Tbox-21 (*t-bet*), novel immune-type receptor 9 (*nitr9*), Natural Killer (NK) cell lysin *(nkl)a, nklb*, *nklc* or *nkld*.

Treatment	Tissue	Upregulated Gene, Hours Post *E. ictaluri* Challenge, Fold Change		
		1–100	101–1000	>1000
beta glucan	Liver		*nkla* (6 h), 250 *nklb* (6 h), 734 *nkld* (6 h), 589	*ifnγ* (6 h), 1369 *nklc* (6 h), 2135
	Kidney	*ifnγ* (24 h), 93 *nkla* (48 h), 2	*ifnγ* (12 h), 575 *nkld* (12 h), 147	*nklb* (6 h), 5536
beta glucan + RE33^®^	Liver		*nkld* (12 h), 108 *nitr9* (24 h), 576 *nkla* (24 h), 332 *nklb* (24 h), 598 *nklc* (24 h), 810 *nkld* (24 h), 246	*ifnγ* (24 h), 1400 *t-bet* (24 h), 3104
	Kidney	*nkla* (48 h), 2	*ifnγ* (48 h), 955 *nkld* (12 h), 165 *nkld* (24 h), 109	
R848 + RE33^®^	Liver		*nitr9* (24 h), 225	*ifnγ* (48 h), 1127
	Kidney	*nkla* (48 h), 6	*nkld* (24 h), 107	
R848	Liver		*nitr9* (12 h), 359	
	Kidney	*nkla* (48 h), 13		
RE33^®^	Liver		*nitr9* (6 h), 284 *nkla* (12 h), 234 *nkld* (12 h), 117	*ifnγ* (48 h), 2133
	Kidney	*nkla* (48 h), 4		*t-bet* (6 h), 280,559
Saline injected, or sham control	Liver	*ifnγ* (12 h), 92 *nklc* (48 h), 71	*ifnγ* (6 h), 101	*ifnγ* (48 h), 1962
	Kidney	*nitr9* (6 h), 10 *nitr9* (12 h), 12 *nitr9* (48 h), 0.4 *nkla* (48 h), 6	*ifnγ* (24 h), 992	

**Table 3:** Oligonucleotide primers and probes used for qRT-PCR to quantify gene expression levels of *arp*, *ifnγ*, *t-bet*, *nitr9*, *nkla*, *nklb*, *nklc and nkld*. Housekeeping gene *arp* was used as a reference gene. * The *t-bet* primers and probe were designed by Beacon Design software (BioRad) and Primer3 plus (GraphPad) software, respectively.

Gene	Oligonucleotide Sequences (5′–3′)	GenBank Accession No.
*arp*	**Fwd:** CTGCAAAGATGCCCAGGGA **Rev:** TTGGAGCCGACATTGTCTGC **Probe:** [6~FAM]TTCTGAAAATCATCCAACTGCTGGATGACTACC [BHQ1a~ Q] [49]	NM_131580
*ifnγ*	**Fwd:** CTTTCCAGGCAAGAGTGCAGA **Rev:** TCAGCTCAAACAAAGCCTTTCG **Probe:** [6~FAM]AACGCTATGGGCGATCAAGGAAAACGAC[BHQ1a~ Q] [49]	NM_212864
*t-bet*	**Fwd:** GATCAAGCTCTCTCTGTGATAG **Rev:** GCTAAAGTCACACAGGTCT **Probe:** [6~FAM]TTCTGAAGGTCACGGTCACA[BHQ1a~Q] *	NM_001170599.1
*nitr9*	**Fwd:** GTCAAAGGGACAAGGCTGATAGTT **Rev:** GTTCAAAACAGTGCATGTAAGACTCA **Probe:** [6~FAM]CAAGGTTTGGAAAAGCAC[BHQ1a~Q] [31]	AY570237.1
*nkla*	**Fwd:** TTTCTGGTCGGCTTGCTCAT **Rev:** TTCTCATTCACAGCCCGGTC **Probe:** [6~FAM]TCTGCAGCTCACTGGGAGGTTCGTGA[BHQ1a~Q]	NM_001311794
*nklb*	**Fwd:** TCCGCAACATCTTTCTGGTCA **Rev:** AGCCTGCTCATGAATGAAAATGA **Probe:** [6~FAM]CACGCCTGCAAATCTGAACCACCCA[BHQ1a~Q]	NM_001311792
*nklc*	**Fwd:** CTGCTTGTGCTGCTCACTTG **Rev:** AGCACACATGGAGATGAGAACA **Probe:** [6~FAM]GGGCTTGCAAGTGGGCCATGGGAA[BHQ1a~Q]	NM_001311793.1
*nkld*	**Fwd:** ACCCTGCTCATCTCCTCTGT **Rev:** CCCCAGCTAAAGCAAAACCC **Probe:** [6~FAM]TGCCTGGGATGTGCTGGGCTTGCAA[BHQ1a~Q]	NM_212741.1

## Data Availability

The data supporting the findings of this paper are available from the corresponding author upon reasonable request.

## Supplementary Materials

The following supporting information can be downloaded at: https://www.mdpi.com/article/10.3390/ijms26030962/s1.
