# Supplementary material for: Rapid Natural Killer Cell Gene Responses, Generated by TLR Ligand-Induced Trained Immunity, Provide Protection to Bacterial Infection in rag1−/− Mutant Zebrafish (Danio rerio)
Source: Int J Mol Sci. 2025 Jan 23;26(3):962. doi: 10.3390/ijms26030962 (PMC11818001; doi:10.3390/ijms26030962)
Supplement: Supplementary file 1 [file ijms-26-00962-s001.zip › ijms-3360724-supplementary/rapid gene response Supp Fig 5 Kidney Correlation Matrix.pdf]

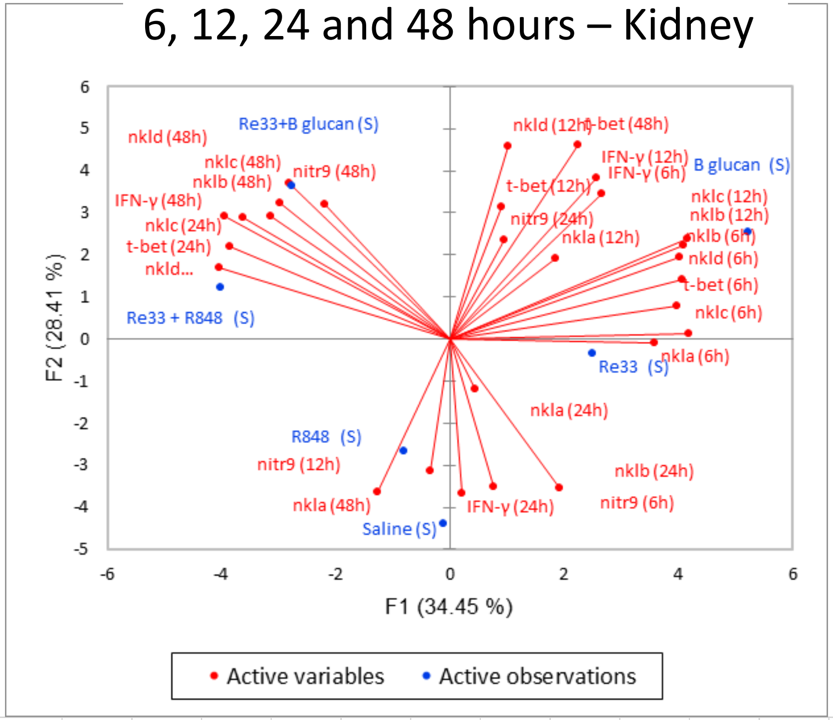

Correlation matrix (Pearson (n)):

Variables	IFN-γ (6h)	IFN-γ (12h)	IFN-γ (24h)	IFN-γ (48h)	nitr9 (6h)	nitr9 (12h)	nitr9 (24h)	nitr9 (48h)	t-bet (6h)	t-bet (12h)	t-bet (24h)	t-bet (48h)	nkla (6h)	nkla (12h)	nkla (24h)	nkla (48h)	nklb (6h)	nklb (12h)	nklb (24h)	nklb (48h)	nklc (6h)	nklc (12h)	nklc (24h)	nklc (48h)	nkld (6h)	nkld (12h)	nkld (24h)	nkld (48h)
IFN-γ (6h)	**1**	0.531	-0.385	0.028	-0.254	-0.496	-0.068	-0.218	0.796	0.033	-0.018	**0.824**	0.726	0.722	-0.151	-0.790	0.453	0.648	-0.288	0.343	0.684	0.604	-0.152	0.113	0.734	0.518	-0.007	0.189
IFN-γ (12h	0.531	**1**	-0.220	-0.162	-0.167	-0.119	0.693	0.456	0.254	**0.824**	-0.288	**0.893**	0.093	0.192	0.254	-0.648	0.764	0.774	0.013	-0.103	0.374	**0.846**	0.027	0.319	0.668	**0.915**	0.093	-0.010
IFN-γ (24h	-0.385	-0.220	**1**	-0.500	**0.989**	**0.953**	-0.293	-0.340	-0.245	-0.304	-0.093	-0.356	-0.035	-0.333	**0.851**	0.019	-0.331	-0.214	**0.915**	-0.461	0.144	-0.222	0.023	-0.204	0.160	-0.280	-0.097	-0.236
IFN-γ (48h	0.028	-0.162	-0.500	**1**	-0.554	-0.423	-0.128	0.497	-0.430	-0.029	**0.894**	0.070	-0.456	-0.026	-0.347	-0.150	-0.501	-0.438	-0.790	**0.924**	-0.571	-0.475	0.790	0.731	-0.513	0.162	**0.841**	**0.875**
nitr9 (6h)	-0.254	-0.167	**0.989**	-0.554	**1**	**0.914**	-0.329	-0.427	-0.108	-0.333	-0.137	-0.268	0.100	-0.236	**0.849**	-0.071	-0.261	-0.114	**0.927**	-0.464	0.276	-0.131	-0.047	-0.247	0.285	-0.247	-0.152	-0.257
nitr9 (12h)	-0.496	-0.119	**0.953**	-0.423	**0.914**	**1**	-0.043	-0.063	-0.414	-0.087	-0.076	-0.332	-0.215	-0.353	**0.865**	0.025	-0.341	-0.299	**0.869**	-0.447	-0.077	-0.256	0.144	-0.007	0.033	-0.121	0.016	-0.238
nitr9 (24h)	-0.068	0.693	-0.293	-0.128	-0.329	-0.043	**1**	0.749	-0.139	**0.938**	-0.411	0.385	-0.291	0.022	-0.006	-0.058	0.554	0.294	-0.058	-0.284	-0.254	0.457	-0.029	0.309	0.029	0.668	-0.021	-0.304
nitr9 (48h)	-0.218	0.456	-0.340	0.497	-0.427	-0.063	0.749	**1**	-0.600	0.789	0.278	0.279	-0.712	-0.214	0.014	-0.114	0.049	-0.110	-0.366	0.272	-0.640	0.004	0.611	0.765	-0.333	0.642	0.611	0.336
t-bet (6h)	0.796	0.254	-0.245	-0.430	-0.108	-0.414	-0.139	-0.600	**1**	-0.184	-0.487	0.448	**0.959**	0.748	-0.291	-0.362	0.534	0.613	-0.028	-0.124	0.785	0.582	-0.703	-0.440	0.675	0.089	-0.593	-0.368
t-bet (12h)	0.033	**0.824**	-0.304	-0.029	-0.333	-0.087	**0.938**	0.789	-0.184	**1**	-0.274	0.548	-0.374	-0.130	0.109	-0.203	0.626	0.456	-0.089	-0.186	-0.129	0.579	0.135	0.380	0.173	0.776	0.150	-0.075
t-bet (24h)	-0.018	-0.288	-0.093	**0.894**	-0.137	-0.076	-0.411	0.278	-0.487	-0.274	**1**	-0.024	-0.412	-0.126	0.012	-0.252	-0.710	-0.523	-0.470	**0.882**	-0.439	-0.597	**0.882**	0.690	-0.394	0.038	**0.899**	**0.934**
t-bet (48h)	**0.824**	**0.893**	-0.356	0.070	-0.268	-0.332	0.385	0.279	0.448	0.548	-0.024	**1**	0.314	0.437	0.105	**-0.859**	0.615	0.744	-0.218	0.250	0.488	0.760	0.115	0.405	0.727	**0.888**	0.231	0.255
nkla (6h)	0.726	0.093	-0.035	-0.456	0.100	-0.215	-0.291	-0.712	**0.959**	-0.374	-0.412	0.314	**1**	0.783	-0.164	-0.372	0.316	0.430	0.112	-0.112	0.747	0.396	-0.661	-0.429	0.628	-0.025	-0.571	-0.375
nkla (12h)	0.722	0.192	-0.333	-0.026	-0.236	-0.353	0.022	-0.214	0.748	-0.130	-0.126	0.437	0.783	**1**	-0.354	-0.508	0.134	0.157	-0.272	0.274	0.283	0.197	-0.314	0.117	0.318	0.297	-0.191	-0.147
nkla (24h)	-0.151	0.254	**0.851**	-0.347	**0.849**	**0.865**	-0.006	0.014	-0.291	0.109	0.012	0.105	-0.164	-0.354	**1**	-0.353	-0.080	0.085	0.799	-0.310	0.215	0.093	0.288	0.135	0.400	0.217	0.201	0.018
nkla (48h)	-0.790	-0.648	0.019	-0.150	-0.071	0.025	-0.058	-0.114	-0.362	-0.203	-0.252	**-0.859**	-0.372	-0.508	-0.353	**1**	-0.195	-0.439	0.047	-0.419	-0.444	-0.432	-0.319	-0.537	-0.683	-0.761	-0.414	-0.411
nklb (6h)	0.453	0.764	-0.331	-0.501	-0.261	-0.341	0.554	0.049	0.534	0.626	-0.710	0.615	0.316	0.134	-0.080	-0.195	**1**	**0.924**	0.050	-0.470	0.589	**0.961**	-0.537	-0.313	0.658	0.472	-0.477	-0.420
nklb (12h)	0.648	0.774	-0.214	-0.438	-0.114	-0.299	0.294	-0.110	0.613	0.456	-0.523	0.744	0.430	0.157	0.085	-0.439	**0.924**	**1**	0.098	-0.313	0.800	**0.981**	-0.408	-0.266	**0.859**	0.497	-0.335	-0.197
nklb (24h)	-0.288	0.013	**0.915**	-0.790	**0.927**	**0.869**	-0.058	-0.366	-0.028	-0.089	-0.470	-0.218	0.112	-0.272	0.799	0.047	0.050	0.098	**1**	-0.750	0.345	0.118	-0.289	-0.415	0.368	-0.179	-0.398	-0.535
nklb (48h)	0.343	-0.103	-0.461	**0.924**	-0.464	-0.447	-0.284	0.272	-0.124	-0.186	**0.882**	0.250	-0.112	0.274	-0.310	-0.419	-0.470	-0.313	-0.750	**1**	-0.303	-0.371	0.692	0.707	-0.253	0.227	0.780	**0.876**
nklc (6h)	0.684	0.374	0.144	-0.571	0.276	-0.077	-0.254	-0.640	0.785	-0.129	-0.439	0.488	0.747	0.283	0.215	-0.444	0.589	0.800	0.345	-0.303	**1**	0.717	-0.524	-0.504	**0.919**	0.105	-0.467	-0.231
nklc (12h)	0.604	**0.846**	-0.222	-0.475	-0.131	-0.256	0.457	0.004	0.582	0.579	-0.597	0.760	0.396	0.197	0.093	-0.432	**0.961**	**0.981**	0.118	-0.371	0.717	**1**	-0.423	-0.207	**0.826**	0.582	-0.353	-0.286
nklc (24h)	-0.152	0.027	0.023	0.790	-0.047	0.144	-0.029	0.611	-0.703	0.135	**0.882**	0.115	-0.661	-0.314	0.288	-0.319	-0.537	-0.408	-0.289	0.692	-0.524	-0.423	**1**	**0.853**	-0.324	0.330	**0.987**	**0.865**
nklc (48h)	0.113	0.319	-0.204	0.731	-0.247	-0.007	0.309	0.765	-0.440	0.380	0.690	0.405	-0.429	0.117	0.135	-0.537	-0.313	-0.266	-0.415	0.707	-0.504	-0.207	**0.853**	**1**	-0.197	0.661	**0.891**	0.689
nkld (6h)	0.734	0.668	0.160	-0.513	0.285	0.033	0.029	-0.333	0.675	0.173	-0.394	0.727	0.628	0.318	0.400	-0.683	0.658	**0.859**	0.368	-0.253	**0.919**	**0.826**	-0.324	-0.197	**1**	0.461	-0.262	-0.154
nkld (12h)	0.518	**0.915**	-0.280	0.162	-0.247	-0.121	0.668	0.642	0.089	0.776	0.038	**0.888**	-0.025	0.297	0.217	-0.761	0.472	0.497	-0.179	0.227	0.105	0.582	0.330	0.661	0.461	**1**	0.408	0.246
nkld (24h)	-0.007	0.093	-0.097	**0.841**	-0.152	0.016	-0.021	0.611	-0.593	0.150	**0.899**	0.231	-0.571	-0.191	0.201	-0.414	-0.477	-0.335	-0.398	0.780	-0.467	-0.353	**0.987**	**0.891**	-0.262	0.408	**1**	**0.909**
nkld (48h)	0.189	-0.010	-0.236	**0.875**	-0.257	-0.238	-0.304	0.336	-0.368	-0.075	**0.934**	0.255	-0.375	-0.147	0.018	-0.411	-0.420	-0.197	-0.535	**0.876**	-0.231	-0.286	**0.865**	0.689	-0.154	0.246	**0.909**	**1**
