# Supplementary material for: Rapid Natural Killer Cell Gene Responses, Generated by TLR Ligand-Induced Trained Immunity, Provide Protection to Bacterial Infection in rag1−/− Mutant Zebrafish (Danio rerio)
Source: Int J Mol Sci. 2025 Jan 23;26(3):962. doi: 10.3390/ijms26030962 (PMC11818001; doi:10.3390/ijms26030962)
Supplement: Supplementary file 1 [file ijms-26-00962-s001.zip › ijms-3360724-supplementary/rapid gene response Supp Fig 6 Liver Correlation Matrix.pdf]

6, 12, 24 and 48 hours – Liver

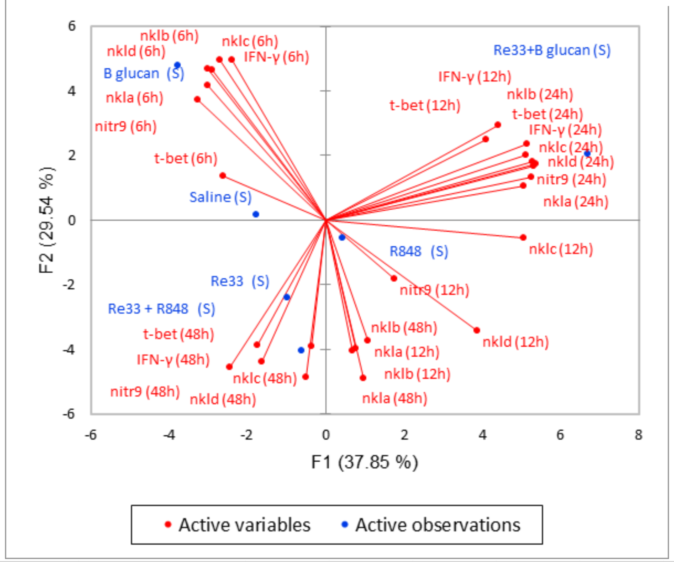

Correlation matrix (Pearson (n)):

Variables	IFN-γ (6h)	IFN-γ (12h)	IFN-γ (24h)	IFN-γ (48h)	nitr9 (6h)	nitr9 (12h)	nitr9 (24h)	nitr9 (48h)	t-bet (6h)	t-bet (12h)	t-bet (24h)	t-bet (48h)	nkla (6h)	nkla (12h)	nkla (24h)	nkla (48h)	nklb (6h)	nklb (12h)	nklb (24h)	nklb (48h)	nklc (6h)	nklc (12h)	nklc (24h)	nklc (48h)	nkld (6h)	nkld (12h)	nkld (24h)	nkld (48h)
IFN-γ (6h)	**1**	0.229	-0.018	-0.428	**0.889**	-0.420	-0.177	-0.362	0.755	-0.008	-0.107	-0.099	**0.979**	-0.190	-0.149	-0.429	**0.991**	-0.211	-0.028	-0.184	**0.994**	-0.406	-0.156	-0.287	**0.992**	-0.487	-0.146	-0.418
IFN-γ (12h	0.229	**1**	**0.935**	-0.297	0.125	-0.145	0.793	-0.589	0.206	0.594	**0.894**	-0.190	0.120	0.337	**0.911**	0.052	0.139	0.263	**0.910**	0.268	0.132	0.539	**0.851**	-0.098	0.107	0.451	**0.874**	-0.320
IFN-γ (24h	-0.018	**0.935**	**1**	-0.399	-0.194	0.019	**0.929**	-0.572	-0.131	0.759	**0.992**	-0.374	-0.152	0.182	**0.978**	-0.001	-0.097	0.107	**0.993**	0.114	-0.117	0.726	**0.977**	-0.103	-0.140	0.520	**0.984**	-0.256
IFN-γ (48h	-0.428	-0.297	-0.399	**1**	0.001	-0.294	-0.348	0.585	0.050	-0.768	-0.367	0.522	-0.346	0.475	-0.229	0.417	-0.456	0.511	-0.419	0.500	-0.395	-0.433	-0.370	0.458	-0.385	0.030	-0.358	0.299
nitr9 (6h)	**0.889**	0.125	-0.194	0.001	**1**	-0.546	-0.391	-0.245	**0.813**	-0.310	-0.271	0.131	**0.902**	-0.026	-0.239	-0.303	**0.877**	-0.027	-0.202	-0.034	**0.889**	-0.625	-0.333	-0.250	**0.897**	-0.557	-0.301	-0.466
nitr9 (12h)	-0.420	-0.145	0.019	-0.294	-0.546	**1**	-0.082	-0.282	-0.321	0.499	0.015	0.313	-0.333	0.120	0.021	0.482	-0.327	0.153	0.008	0.033	-0.428	0.680	0.004	-0.408	-0.426	0.514	0.054	0.041
nitr9 (24h)	-0.177	0.793	**0.929**	-0.348	-0.391	-0.082	**1**	-0.280	-0.303	0.639	**0.947**	-0.507	-0.326	0.097	**0.898**	-0.060	-0.270	0.018	**0.925**	0.085	-0.254	0.662	**0.966**	0.179	-0.281	0.506	**0.934**	0.013
nitr9 (48h)	-0.362	-0.589	-0.572	0.585	-0.245	-0.282	-0.280	**1**	-0.036	-0.786	-0.541	0.259	-0.285	0.193	-0.545	0.247	-0.385	0.219	-0.606	0.339	-0.273	-0.466	-0.490	**0.858**	-0.279	-0.010	-0.561	**0.851**
t-bet (6h)	0.755	0.206	-0.131	0.050	**0.813**	-0.321	-0.303	-0.036	**1**	-0.334	-0.246	0.522	**0.836**	0.458	-0.200	0.217	0.727	0.454	-0.204	0.473	0.765	-0.375	-0.320	0.075	0.753	-0.056	-0.292	-0.016
t-bet (12h)	-0.008	0.594	0.759	-0.768	-0.310	0.499	0.639	-0.786	-0.334	**1**	0.756	-0.438	-0.109	-0.197	0.695	-0.134	0.003	-0.241	0.783	-0.299	-0.089	**0.835**	0.749	-0.551	-0.099	0.358	0.773	-0.430
t-bet (24h)	-0.107	**0.894**	**0.992**	-0.367	-0.271	0.015	**0.947**	-0.541	-0.246	0.756	**1**	-0.439	-0.251	0.121	**0.983**	-0.043	-0.184	0.047	**0.996**	0.056	-0.204	0.726	**0.995**	-0.093	-0.226	0.496	**0.998**	-0.253
t-bet (48h)	-0.099	-0.190	-0.374	0.522	0.131	0.313	-0.507	0.259	0.522	-0.438	-0.439	**1**	0.090	0.792	-0.312	**0.857**	-0.073	**0.839**	-0.457	0.772	-0.066	-0.057	-0.498	0.169	-0.070	0.416	-0.441	0.341
nkla (6h)	**0.979**	0.120	-0.152	-0.346	**0.902**	-0.333	-0.326	-0.285	**0.836**	-0.109	-0.251	0.090	**1**	-0.084	-0.277	-0.283	**0.983**	-0.091	-0.175	-0.077	**0.986**	-0.442	-0.308	-0.263	**0.985**	-0.443	-0.290	-0.335
nkla (12h)	-0.190	0.337	0.182	0.475	-0.026	0.120	0.097	0.193	0.458	-0.197	0.121	0.792	-0.084	**1**	0.241	**0.902**	-0.242	**0.996**	0.083	**0.985**	-0.202	0.252	0.067	0.421	-0.225	0.752	0.103	0.421
nkla (24h)	-0.149	**0.911**	**0.978**	-0.229	-0.239	0.021	**0.898**	-0.545	-0.200	0.695	**0.983**	-0.312	-0.277	0.241	**1**	0.063	-0.228	0.173	**0.976**	0.160	-0.251	0.713	**0.968**	-0.102	-0.270	0.541	**0.985**	-0.279
nkla (48h)	-0.429	0.052	-0.001	0.417	-0.303	0.482	-0.060	0.247	0.217	-0.134	-0.043	**0.857**	-0.283	**0.902**	0.063	**1**	-0.431	**0.923**	-0.091	**0.878**	-0.422	0.379	-0.082	0.323	-0.439	0.811	-0.043	0.520
nklb (6h)	**0.991**	0.139	-0.097	-0.456	**0.877**	-0.327	-0.270	-0.385	0.727	0.003	-0.184	-0.073	**0.983**	-0.242	-0.228	-0.431	**1**	-0.253	-0.101	-0.244	**0.990**	-0.408	-0.233	-0.370	**0.992**	-0.523	-0.218	-0.448
nklb (12h)	-0.211	0.263	0.107	0.511	-0.027	0.153	0.018	0.219	0.454	-0.241	0.047	**0.839**	-0.091	**0.996**	0.173	**0.923**	-0.253	**1**	0.009	**0.980**	-0.217	0.219	-0.007	0.404	-0.238	0.729	0.032	0.428
nklb (24h)	-0.028	**0.910**	**0.993**	-0.419	-0.202	0.008	**0.925**	-0.606	-0.204	0.783	**0.996**	-0.457	-0.175	0.083	**0.976**	-0.091	-0.101	0.009	**1**	0.011	-0.128	0.708	**0.986**	-0.162	-0.148	0.446	**0.993**	-0.325
nklb (48h)	-0.184	0.268	0.114	0.500	-0.034	0.033	0.085	0.339	0.473	-0.299	0.056	0.772	-0.077	**0.985**	0.160	**0.878**	-0.244	**0.980**	0.011	**1**	-0.183	0.166	0.011	0.563	-0.208	0.717	0.030	0.550
nklc (6h)	**0.994**	0.132	-0.117	-0.395	**0.889**	-0.428	-0.254	-0.273	0.765	-0.089	-0.204	-0.066	**0.986**	-0.202	-0.251	-0.422	**0.990**	-0.217	-0.128	-0.183	**1**	-0.474	-0.249	-0.237	**0.999**	-0.523	-0.245	-0.351
nklc (12h)	-0.406	0.539	0.726	-0.433	-0.625	0.680	0.662	-0.466	-0.375	**0.835**	0.726	-0.057	-0.442	0.252	0.713	0.379	-0.408	0.219	0.708	0.166	-0.474	**1**	0.719	-0.201	-0.494	0.792	0.744	-0.004
nklc (24h)	-0.156	**0.851**	**0.977**	-0.370	-0.333	0.004	**0.966**	-0.490	-0.320	0.749	**0.995**	-0.498	-0.308	0.067	**0.968**	-0.082	-0.233	-0.007	**0.986**	0.011	-0.249	0.719	**1**	-0.054	-0.270	0.476	**0.994**	-0.214
nklc (48h)	-0.287	-0.098	-0.103	0.458	-0.250	-0.408	0.179	**0.858**	0.075	-0.551	-0.093	0.169	-0.263	0.421	-0.102	0.323	-0.370	0.404	-0.162	0.563	-0.237	-0.201	-0.054	**1**	-0.262	0.286	-0.132	**0.876**
nkld (6h)	**0.992**	0.107	-0.140	-0.385	**0.897**	-0.426	-0.281	-0.279	0.753	-0.099	-0.226	-0.070	**0.985**	-0.225	-0.270	-0.439	**0.992**	-0.238	-0.148	-0.208	**0.999**	-0.494	-0.270	-0.262	**1**	-0.553	-0.265	-0.374
nkld (12h)	-0.487	0.451	0.520	0.030	-0.557	0.514	0.506	-0.010	-0.056	0.358	0.496	0.416	-0.443	0.752	0.541	0.811	-0.523	0.729	0.446	0.717	-0.523	0.792	0.476	0.286	-0.553	**1**	0.494	0.439
nkld (24h)	-0.146	**0.874**	**0.984**	-0.358	-0.301	0.054	**0.934**	-0.561	-0.292	0.773	**0.998**	-0.441	-0.290	0.103	**0.985**	-0.043	-0.218	0.032	**0.993**	0.030	-0.245	0.744	**0.994**	-0.132	-0.265	0.494	**1**	-0.281
nkld (48h)	-0.418	-0.320	-0.256	0.299	-0.466	0.041	0.013	**0.851**	-0.016	-0.430	-0.253	0.341	-0.335	0.421	-0.279	0.520	-0.448	0.428	-0.325	0.550	-0.351	-0.004	-0.214	**0.876**	-0.374	0.439	-0.281	**1**

Correlation matrix (Pearson (n)):
