# Supplementary material for: Rapid Natural Killer Cell Gene Responses, Generated by TLR Ligand-Induced Trained Immunity, Provide Protection to Bacterial Infection in rag1−/− Mutant Zebrafish (Danio rerio)
Source: Int J Mol Sci. 2025 Jan 23;26(3):962. doi: 10.3390/ijms26030962 (PMC11818001; doi:10.3390/ijms26030962)
Supplement: Supplementary file 1 [file ijms-26-00962-s001.zip › ijms-3360724-supplementary/rapid gene response Supp Fig 7 kidney FC.pdf]

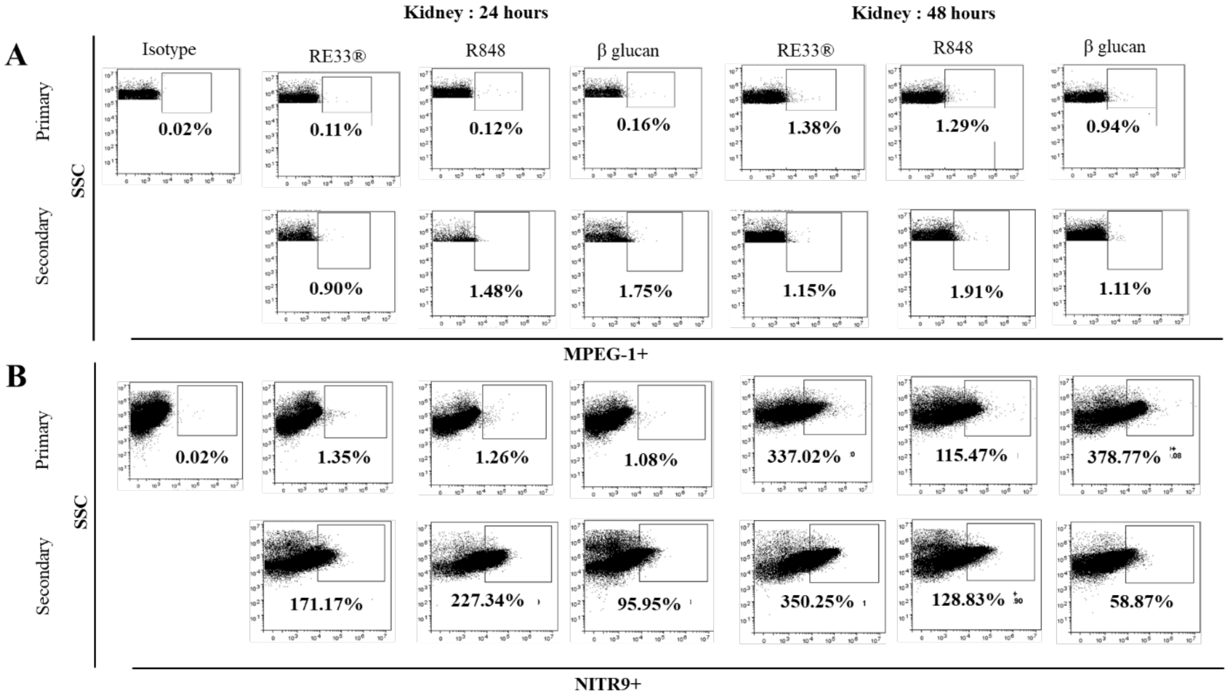

Supplemental Figure 7: The flow cytometry dot plots showing the mean percentage of cell counts for primary and secondary exposures. (A) MPEG-1+ kidney leukocytes at 24 and 48 hours (h) after exposure to RE33®, R848, or β glucan IC injections (n=15 per group), and (B) NITR9+ kidney leukocytes at 24 and 48 hours after exposure to RE33®, R848, or β glucan IC injections (n=15 per group). The gating strategy involved the following steps: 1. Cells were visualized as dot plots on forward scatter (FSC) and side scatter (SSC) graphs. 2. Flow cytometry gating was performed by drawing a gate around the leukocytes. 3. Live cells were selected while dead cells were excluded. 4. A subsequent gate was drawn around singlet cells, excluding doublet cells. 5. Cells were plotted as dot plots on SSC and APC for MPEG+ cells and on SSC and PE for NITR9+ leukocytes and MPEG+ and NITR9+ cells. The percentages of MPEG+ and NITR9+ cells are reported in representative dot plots, and mean cell counts are shown in the bar graphs. Appropriate isotype controls were utilized to eliminate any nonspecific staining.
