# Supplementary material for: Rapid Natural Killer Cell Gene Responses, Generated by TLR Ligand-Induced Trained Immunity, Provide Protection to Bacterial Infection in rag1−/− Mutant Zebrafish (Danio rerio)
Source: Int J Mol Sci. 2025 Jan 23;26(3):962. doi: 10.3390/ijms26030962 (PMC11818001; doi:10.3390/ijms26030962)
Supplement: Supplementary file 1 [file ijms-26-00962-s001.zip › ijms-3360724-supplementary/rapid gene response Supplemental Figure Captions.pdf]

Supplemental Figure Captions (1-8)

Figure 1: Trained immunity was induced by IP injection of PBS (control), R848, R848+RE33®, β glucan+RE33®, β glucan and RE33®. After 1 month, each treatment group was challenged by IP injection of WT *E. ictaluri.* Quantitative analysis of *rag1*$^{-/-}$ mutant zebrafish kidney (A) *Ifnγ*, (B) *Nitr9* and (C) *T-bet* by qPCR was performed at 6, 12, 24 and 48 hours post injection (hpi). The transcript levels of each gene were normalized to that of *Arp* (house-keeping gene) and fold changes were calculated and reported. Each bar represents the mean of the CT values from the livers of three biological replicates. Each biological replicate represents a kidney from an individual zebrafish. *Statistical significance was analyzed between the fish that received PBS mock-immunization and the fish that were trained and later challenged (p value < 0.005).

Figure 2: Trained immunity was induced by IP injection of PBS (control), R848, R848+RE33®, β glucan+RE33®, β glucan and RE33®. After 1 month, each treatment group was challenged by IP injection of WT *E. ictaluri*. Quantitative analysis of *rag1*$^{-/-}$ mutant zebrafish kidney (A) *Nkla*, (B) *Nklb*, (C) *Nklc* and (D) *Nkld* by qPCR were performed at 6, 12, 24 and 48 hours post injection (hpi). The transcript levels of each gene were normalized to that of *Arp* (house-keeping gene) and fold changes were calculated and reported. Each bar represents the mean of the CT values from the kidneys of three biological replicates. Each biological replicate represents a kidney from an individual zebrafish. *Statistical significance was analyzed between the fish that received PBS mock-immunization and the fish that were trained and later challenged (p value < 0.005).

Figure 3: Trained immunity was induced by IP injection of PBS (control), R848, R848+RE33®, β glucan+RE33®, β glucan and RE33®. After 1 month, each treatment group was challenged by IP injection of WT *E. ictaluri.* Quantitative analysis of *rag1*$^{-/-}$ mutant zebrafish liver (A) *Ifnγ*, (B) *Nitr9* and (C) *T-bet* by qPCR were performed at 6, 12, 24 and 48 hours post injection (hpi). The transcript levels of each gene were normalized to that of *Arp* (house-keeping gene) and fold changes were calculated and reported. Each bar represents the mean of the CT values from the livers of three biological replicates. Each biological replicate represents a liver from an individual zebrafish. *Statistical significance was analyzed between the fish that received PBS mock-immunization and the fish that were trained and later challenged (p value < 0.005).

Figure 4: Trained immunity was induced by IP injection of PBS (control), R848, R848+RE33®, β glucan+RE33®, β glucan and RE33®. After 1 month, each treatment group was challenged by IP injection of WT *E. ictaluri.* Quantitative analysis of *rag1*$^{-/-}$ mutant zebrafish liver (A) *Nkla*, (B) *Nklb*, (C) *Nklc* and (D) *Nkld* by qPCR were performed at 6, 12, 24 and 48 hours post injection (hpi). The transcript levels of each gene were normalized to that of *Arp* (house-keeping gene) and fold changes were calculated and reported. Each bar represents the mean of the CT values from the livers of three biological replicates. Each biological replicate represents a liver from an individual zebrafish. *Statistical significance was analyzed between the fish that received PBS mock-immunization and the fish that were trained and later challenged (p value < 0.005).

Figure 5. Pearson correlation matrix and Principal Component Analysis (PCA) biplot summarizing the relationships between kidney NK cell immune genes at 6, 12, 24 and 48 hours post bacterial exposure, and the associations of the gene relationships with survival.

Figure 6. Pearson correlation matrix and Principal Component Analysis (PCA) biplot summarizing the relationships between liver NK cell immune genes at 6, 12, 24 and 48 hours post bacterial exposure, and the associations of the gene relationships with survival.

Figure 7. Representative flow cytometry dot plots of *rag1*$^{-/-}$ mutant zebrafish kidney leukocytes after exposure to the TLR ligand or RE33® (primary) and after challenge 1 month later with WT *E. ictaluri* (secondary). (A) MPEG-1^{+} liver leukocytes at 24 and 48 hours (h) after exposure to RE33®, R848, or β glucan IC injections (n=15 per group), and (B) NITR9^{+} liver leukocytes at 24 and 48 hours after exposure to RE33®, R848, or β glucan IC injections (n=15 per group). The gating strategy involved the following steps: 1. Cells were visualized as dot plots on forward scatter (FSC) and side scatter (SSC) graphs. 2. Flow cytometry gating was performed by drawing a gate around the leukocytes. 3. Live cells were selected while dead cells were excluded. 4. A subsequent gate was drawn around singlet cells, excluding doublet cells. 5. Cells were plotted as dot plots on SSC and APC for MPEG^{+} cells and on SSC and PE for NITR9^{+} leukocytes and MPEG+ and NITR9+ cells. The percentages of MPEG+ and NITR9+ cells are reported in representative dot plots. Appropriate isotype controls were utilized to eliminate any nonspecific staining.

Figure 8. Representative flow cytometry dot plots of *rag1*$^{-/-}$ mutant zebrafish liver leukocytes after exposure to the TLR ligand or RE33® (primary) and after challenge 1 month later with WT *E. ictaluri* (secondary). (A) MPEG-1^{+} liver leukocytes at 24 and 48 hours (h) after exposure to RE33®, R848, or β glucan IC injections (n=15 per group), and (B) NITR9^{+} liver leukocytes at 24 and 48 hours after exposure to RE33®, R848, or β glucan IC injections (n=15 per group). The gating strategy involved the following steps: 1. Cells were visualized as dot plots on forward scatter (FSC) and side scatter (SSC) graphs. 2. Flow cytometry gating was performed by drawing a gate around the leukocytes. 3. Live cells were selected while dead cells were excluded. 4. A subsequent gate was drawn around singlet cells, excluding doublet cells. 5. Cells were plotted as dot plots on SSC and APC for MPEG^{+} cells and on SSC and PE for NITR9^{+} leukocytes and MPEG^{+} and NITR9^{+} cells. The percentages of MPEG^{+} and NITR9^{+} cells are reported in representative dot plots. Appropriate isotype controls were utilized to eliminate any nonspecific staining.
