# Supplementary material for: Rapid Natural Killer Cell Gene Responses, Generated by TLR Ligand-Induced Trained Immunity, Provide Protection to Bacterial Infection in rag1−/− Mutant Zebrafish (Danio rerio)
Source: Int J Mol Sci. 2025 Jan 23;26(3):962. doi: 10.3390/ijms26030962 (PMC11818001; doi:10.3390/ijms26030962)
Supplement: Supplementary file 1 [file ijms-26-00962-s001.zip › ijms-3360724-supplementary/Table S1 gene expression ANOVA.pdf]

Supplemental Table S1: ANOVA analysis of gene expression (fold changes). Gray highlighted entries designate values that were significantly different from time 0.

Treatment	Gene	Tissue	Time	Mean ± SD (Challenge)	Mean ± SD (PBS)
**Sham vaccination**	***Ifnγ***	Liver	6h	101.15±10.91 (0.0172)	34.16±21.18
			12h	91.87±91.2 (0.0056)	1531.35±2009.44
			24h	36.82±17.08	2.05±0.75
			48h	1961.90±303.72 (0.0065)	543.08±502.89
		Kidney	6h	29.43±13.27	29.13±11.36
			12h	28.05±1.75	118.05±49.41
			24h	992.04±142.99 (0.0001)	14.72±7.22
			48h	15.85±11.27	5.00±2.07
	T-bet	Liver	6h	344.85±94.31	329.56±252.22
			12h	72.70±16.52	38.98±50.43
			24h	76.32±44.75	7.74±10.01
			48h	147.56±179.56	3479.65±1030.15
		Kidney	6h	49065.40±23728.97	113274.49±75465.53
			12h	23551.85±13505.67	5510.68±2292.01
			24h	42372.51±24723.35	49539.49±46201.41
			48h	22819.74±4531.83	37631.19±65706.75
	Nitr9	Liver	6h	290.43±158.53	33.37±31.31
			12h	72.78±63.63	28.77±1.85
			24h	36.64±7.68	2.14±1.85
			48h	82.12±48.10	948.39±284.44
		Kidney	6h	9.51±7.45 (0.0134)	1.41±0.94
			12h	12.07±8.30 (0.0001)	0.08±0.03
			24h	0.57±0.73	1.12±1.56
			48h	0.35±0.33 (0.0005)	0.14±0.14
	Nkla	Liver	6h	13.03±6.80	10.75±5.98
			12h	3.23±1.52	0.37±0.40
			24h	40.74±51.84	6.19±4.15
			48h	11.38±7.00	298.480±322.20 (0.0019)
		Kidney	6h	13.59±1.53	13.14±9.95
			12h	7.41±2.80	0.01±0.02
			24h	12.28±1.13	2.14±1.69
			48h	5.99±3.81 (0.0001)	3.07±2.12
	Nklb	Liver	6h	67.32±19.22	17.11±13.14

			12h	0.20±0.17	0.52±0.90
			24h	22.23±11.09	22.51±24.92
			48h	27.45±5.29	227.03±200.91
		Kidney	6h	20.36±4.91	21.97±10.06
			12h	2.82±1.23	1.38±1.41
			24h	1463.84±441.32	2335.70±3036.07
			48h	761.72±714.97	234.37±211.06
	Nklc	Liver	6h	200.98±64.57	168.99±183.16
			12h	36.08±8.60	1.94±2.18
			24h	84.63±74.10	5.57±3.58
			48h	71.03±31.95<br>(0.0006)	9.95±4.84
		Kidney	6h	179.12±73.53	283.95±161.04
			12h	58.21±24.66	0.02±0.02
			24h	78.23±37.45	40.85±6.03
			48h	49.23±26.49	104.72±29.51
	Nkld	Liver	6h	52.83±24.31	32.28±10.54
			12h	11.26±8.83	1.22±0.88
			24h	20.48±14.36	6.37±4.04
			48h	13.22±6.74	612.19±176.53
		Kidney	6h	116.80±40.62	155.22±80.33
			12h	35.37±21.81	0.85±0.31
			24h	49.75±9.56	6.90±1.90
			48h	31.72±20.90	52.76±20.32
**RE33®**	***Ifnγ***	Liver	6h	160.27±153.07	34.16±21.18
			12h	579.04±413.99	1531.35±2009.44
			24h	246.11±95.84	2.05±0.75
			48h	2132.95±774.88<br>(0.0019)	543.08±502.89
		Kidney	6h	104.10±39.13	29.13±11.36
			12h	50.22±34.05	118.05±49.41
			24h	25.52±9.37	14.72±7.22
			48h	328.19±161.69	5.00±2.07
	T-bet	Liver	6h	1649.27±1507.74	329.56±252.22
			12h	21.40±17.95	38.98±50.43
			24h	218.60±29.45	7.74±10.01
			48h	446.78±179.79	3479.65±1030.15
		Kidney	6h	280550±275127<br>(0.0141)	113275±75466
			12h	4937.97±2163.96	5510.68±2292.01
			24h	35912±10421	49540±46201
			48h	39919.68±4531.83	37631.19±65706.75
	Nitr9	Liver	6h	284.22±198.34	33.37±31.31

				(0.0210)	
			12h	174.05±137.81	28.77±1.85
			24h	78.88±56.57	2.14±1.85
			48h	96.87±4.23	948.39±284.44
		Kidney	6h	1.32±0.54	1.41±0.94
			12h	0.19±0.18	0.08±0.03
			24h	0.54±0.43	1.12±1.56
			48h	0.21±0.21	0.14±0.14
	Nkla	Liver	6h	59.39±45.37	10.75±5.98
			12h	234.09±197.54 (0.0153)	0.37±0.40
			24h	74.23±22.38	6.19±4.15
			48h	173.17±138.55	298.480±322.20
		Kidney	6h	46.71±37.14	13.14±9.95
			12h	98.68±58.01	0.01±0.02
			24h	2.00±1.71	2.14±1.69
			48h	4.31±1.68 (0.0001)	3.07±2.12
	Nklb	Liver	6h	75.62±69.43	17.11±13.14
			12h	297.35±230.11	0.52±0.90
			24h	34.41±6.09	22.51±24.92
			48h	304.65±279.99	227.03±200.91
		Kidney	6h	1585.37±1052.55	21.97±10.06
			12h	2296.84±897.65	1.38±1.41
			24h	347.99±12.87	2335.70±3036.07
			48h	1641.89±1423.73	234.37±211.06
	Nklc	Liver	6h	303.95±212.73	168.99±183.16
			12h	155.90±104.01	1.94±2.18
			24h	71.37±13.17	5.57±3.58
			48h	173.15±103.17	9.95±4.84
		Kidney	6h	221.14±103.08	283.95±161.04
			12h	114.87±80.51	0.02±0.02
			24h	30.26±3.09	40.85±6.03
			48h	34.59±33.05	104.72±29.51
	Nkld	Liver	6h	60.62±33.67	32.28±10.54
			12h	117.22±41.32 (0.0107)	1.22±0.88
			24h	21.89±6.99	6.37±4.04
			48h	82.95±118.05	612.19±176.53
		Kidney	6h	125.16±63.21	155.22±80.33
			12h	49.21±37.11	0.85±0.31
			24h	17.45±9.77	6.90±1.90
			48h	26.43±16.51	52.76±20.32
**β glucan**	***Ifnγ***	Liver	6h	1369.23±985.77	34.16±21.18

				(0.0116)	
			12h	580±833.21	1531.35±2009.44
			24h	208.38±183.73	2.05±0.75
			48h	238.60±135.71	543.08±502.89
		Kidney	6h	100.94±80.85	29.13±11.36
			12h	575.15±299.31 (0.0276)	118.05±49.41
			24h	92.66±48.02 (0.0001)	14.72±7.22
			48h	39.86±28.72	5.00±2.07
	T-bet	Liver	6h	1954.90±710.20	329.56±252.22
			12h	298.29±199.05	38.98±50.43
			24h	66.85±57.53	7.74±10.01
			48h	179.49±179.79	3479.65±1030.15
		Kidney	6h	200692±94460	113274.49±75465.53
			12h	117301±41163	5510.68±2292.01
			24h	20607.28±15163.86	49539.49±46201.41
			48h	65882.08±4531.83	37631.19±65706.75
	Nitr9	Liver	6h	591.12±239.39	33.37±31.31
			12h	63.00±39.99	28.77±1.85
			24h	50.33±44.41	2.14±1.85
			48h	62.15±60.98	948.39±284.44
		Kidney	6h	1.80±0.79	1.41±0.94
			12h	1.80±1.41	0.08±0.03
			24h	1.62±1.18	1.12±1.56
			48h	0.56±0.26	0.14±0.14
	Nkla	Liver	6h	249.51±208.10 (0.0127)	10.75±5.98
			12h	24.01±16.93	0.37±0.40
			24h	8.23±7.50	6.19±4.15
			48h	5.25±2.85	298.480±322.20 (0.0015)
		Kidney	6h	23.92±7.16	13.14±9.95
			12h	31.73±11.21	0.01±0.02
			24h	7.47±2.08	2.14±1.69
			48h	2.19±1.50 (0.0001)	3.07±2.12
	Nklb	Liver	6h	734.20±430.87 (0.0244)	17.11±13.14
			12h	17.52±7.59	0.52±0.90
			24h	58.00±50.72	22.51±24.92
			48h	62.06±47.68	227.03±200.91
		Kidney	6h	5536.22±2044.37 (0.0244)	21.97±10.06
			12h	9866.47±11062.12	1.38±1.41

			24h	719.59±97.27	2335.70±3036.07
			48h	836.02±513.59	234.37±211.06
	Nklc	Liver	6h	2134.91±896.55 (0.0001)	168.99±183.16
			12h	40.47±24.94	1.94±2.18
			24h	44.21±33.94	5.57±3.58
			48h	60.59±26.64	9.95±4.84
		Kidney	6h	279.94±59.11	283.95±161.04
			12h	311.62±108.45	0.02±0.02
			24h	50.06±35.28	40.85±6.03
			48h	49.49±39.27	104.72±29.51
	Nkld	Liver	6h	588.73±81.86 (0.0001)	32.28±10.54
			12h	14.74±13.28	1.22±0.88
			24h	5.17±3.92	6.37±4.04
			48h	18.97±10.26	612.19±176.53 (0.0107)
		Kidney	6h	194.29±64.14	155.22±80.33
			12h	147.11±44.21 (0.0008)	0.85±0.31
			24h	34.58±15.46	6.90±1.90
			48h	34.21±25.26	52.76±20.32
**β glucan +RE33®**	***Ifnγ***	Liver	6h	237.16±64.05	34.16±21.18
			12h	1539.20±273.06	1531.35±2009.44
			24h	1400.53±214.21 (0.0075)	2.05±0.75
			48h	322.96±88.11	543.08±502.89
		Kidney	6h	62.54±32.10	29.13±11.36
			12h	357.19±174.53	118.05±49.41
			24h	46.25±19.48	14.72±7.22
			48h	954.75±859.15 (0.0001)	5.00±2.07
	T-bet	Liver	6h	505.14±130.43	329.56±252.22
			12h	948.25±455.48	38.98±50.43
			24h	3103.95±2467.42 (0.0001)	7.74±10.01
			48h	80.60±179.79	3479.65±1030.15
		Kidney	6h	29171.11±20790.32	113274.49±75465.53
			12h	112173±87112	5510.68±2292.01
			24h	60893.41±6130.78	49539.49±46201.41
			48h	51462.46±4531.83	37631.19±65706.75
	Nitr9	Liver	6h	142.49±18.28	33.37±31.31
			12h	150.94±18.58	28.77±1.85
			24h	576.44±200.12 (0.0001)	2.14±1.85
			48h	45.93±19.60	948.39±284.44

		Kidney	6h	0.43±0.32	1.41±0.94
			12h	4.11±3.25	0.08±0.03
			24h	1.89±0.16	1.12±1.56
			48h	0.97±0.68	0.14±0.14
	Nkla	Liver	6h	17.05±2.89	10.75±5.98
			12h	83.47±34.86	0.37±0.40
			24h	332.07±142.16 (0.0004)	6.19±4.15
			48h	51.54±46.31	298.480±322.20 (0.0015)
		Kidney	6h	2.76±2.39	13.14±9.95
			12h	53.01±38.13	0.01±0.02
			24h	6.42±1.93	2.14±1.69
			48h	2.16±0.96 (0.0001)	3.07±2.12
	Nklb	Liver	6h	88.29±36.06	17.11±13.14
			12h	80.67±8.05	0.52±0.90
			24h	597.75±537.69 (0.0002)	22.51±24.92
			48h	115.47±51.41	227.03±200.91
		Kidney	6h	950.50±442.02	21.97±10.06
			12h	41.46±17.56	1.38±1.41
			24h	236.61±179.50	2335.70±3036.07
			48h	2086.55±474.26	234.37±211.06
	Nklc	Liver	6h	249.35±134.88	168.99±183.16
			12h	320.63±41.23	1.94±2.18
			24h	809.95±551.60 (0.0002)	5.57±3.58
			48h	89.78±44.71	9.95±4.84
		Kidney	6h	64.06±37.23	283.95±161.04
			12h	88.86±35.78	0.02±0.02
			24h	122.05±93.18	40.85±6.03
			48h	209.49±131.08	104.72±29.51
	Nkld	Liver	6h	41.28±4.17	32.28±10.54
			12h	108.03±1.63 (0.0216)	1.22±0.88
			24h	246.20±119.84 (0.0001)	6.37±4.04
			48h	26.60±15.38	612.19±176.53 (0.0015)
		Kidney	6h	79.17±35.66	155.22±80.33
			12h	165.04±106.38 (0.0002)	0.85±0.31
			24h	109.00±80.96 (0.0299)	6.90±1.90
			48h	73.31±26.75	52.76±20.32
**R848**	***Ifnγ***	Liver	6h	38.89±17.43	34.16±21.18

			12h	0.32±0.14	1531.35±2009.44
			24h	138.14±85.33	2.05±0.75
			48h	269.98±148.81	543.08±502.89
		Kidney	6h	7.22±3.83	29.13±11.36
			12h	2.16±0.73	118.05±49.41 (0.0029)
			24h	39.20±10.92	14.72±7.22
			48h	291.81±96.43	5.00±2.07
	T-bet	Liver	6h	314.34±75.17	329.56±252.22
			12h	687.51±261.57	38.98±50.43
			24h	133.21±79.68	7.74±10.01
			48h	233.83±179.79	3479.65±1030.15 (0.0456)
		Kidney	6h	43092.01±22030.56	113274.49±75465.53
			12h	74564.99±50454.61	5510.68±2292.01
			24h	24256.95±3904.60	49539.49±46201.41
			48h	14272.79±4531.83	37631.19±65706.75
	Nitr9	Liver	6h	70.61±12.38	33.37±31.31
			12h	359.10±114.05 (0.0013)	28.77±1.85
			24h	49.46±28.35	2.14±1.85
			48h	63.51±26.45	948.39±284.44
		Kidney	6h	0.08±0.04	1.41±0.94
			12h	2.38±1.29	0.08±0.03
			24h	1.51±0.23	1.12±1.56
			48h	0.61±0.22	0.14±0.14
	Nkla	Liver	6h	23.86±22.14	10.75±5.98
			12h	26.77±8.38	0.37±0.40
			24h	19.86±27.15	6.19±4.15
			48h	82.13±33.52	298.480±322.20 (0.0284)
		Kidney	6h	1.45±0.78	13.14±9.95
			12h	2.92±1.07	0.01±0.02
			24h	2.24±1.08	2.14±1.69
			48h	13.45±0.29 (0.0001)	3.07±2.12
	Nklb	Liver	6h	93.52±85.30	17.11±13.14
			12h	34.05±15.78	0.52±0.90
			24h	38.94±26.06	22.51±24.92
			48h	47.85±21.11	227.03±200.91
		Kidney	6h	2051.52±1329.96	21.97±10.06
			12h	211.93±62.01	1.38±1.41
			24h	475.24±289.39	2335.70±3036.07
			48h	666.44±379.35	234.37±211.06

	Nklc	Liver	6h	126.60±29.15	168.99±183.16
			12h	257.44±186.13	1.94±2.18
			24h	89.55±36.92	5.57±3.58
			48h	15.59±6.29	9.95±4.84
		Kidney	6h	81.78±49.60	283.95±161.04
			12h	79.12±35.94	0.02±0.02
			24h	43.19±21.08	40.85±6.03
			48h	14.88±7.77	104.72±29.51
	Nkld	Liver	6h	21.44±7.60	32.28±10.54
			12h	79.45±34.81	1.22±0.88
			24h	25.62±15.75	6.37±4.04
			48h	44.29±27.75	612.19±176.53
		Kidney	6h	37.50±33.06	155.22±80.33 (0.0091)
			12h	17.53±3.19	0.85±0.31
			24h	17.09±12.57	6.90±1.90
			48h	5.71±1.15	52.76±20.32
**R848 + RE33®**	***Ifnγ***	Liver	6h	46.13±2.22	34.16±21.18
			12h	18.51±4.42	1531.35±2009.44 (0.0033)
			24h	106.40±111.26	2.05±0.75
			48h	1126.93±445.83 (0.0001)	543.08±502.89
		Kidney	6h	62.72±26.77	29.13±11.36
			12h	2.24±0.79	118.05±49.41
			24h	0.21±0.04	14.72±7.22
			48h	1210.85±336.08 (0.0001)	5.00±2.07
	T-bet	Liver	6h	499.89±241.82	329.56±252.22
			12h	65.78±15.98	38.98±50.43
			24h	141.87±104.05	7.74±10.01
			48h	139.96±179.79	3479.65±1030.15
		Kidney	6h	41416.10±13932.68	113274.49±75465.53
			12h	24955.69±5560.68	5510.68±2292.01
			24h	80945.91±26099.41	49539.49±46201.41
			48h	35149.17	37631.19±65706.75
	Nitr9	Liver	6h	80.36±24.50	33.37±31.31
			12h	89.20±43.84	28.77±1.85
			24h	225.29±277.85 (0.0489)	2.14±1.85
			48h	129.12±44.74	948.39±284.44
		Kidney	6h	0.19±0.11	1.41±0.94
			12h	0.14±0.00	0.08±0.03

			24h	0.25±0.05	1.12±1.56
			48h	0.54±0.23	0.14±0.14
	Nkla	Liver	6h	7.90±2.59	10.75±5.98
			12h	44.41±23.96	0.37±0.40
			24h	3.32±3.24	6.19±4.15
			48h	50.83±23.92	298.480±322.20 (0.0091)
		Kidney	6h	3.22±0.58	13.14±9.95
			12h	5.53±5.06	0.01±0.02
			24h	4.27±0.61	2.14±1.69
			48h	5.66±3.85 (0.0001)	3.07±2.12
	Nklb	Liver	6h	8.73±12.12	17.11±13.14
			12h	44.42±3.75	0.52±0.90
			24h	9.15±9.85	22.51±24.92
			48h	116.00±76.89	227.03±200.91
		Kidney	6h	8.76±7.19	21.97±10.06
			12h	596.48±368.12	1.38±1.41
			24h	9.39±7.05	2335.70±3036.07
			48h	2470.30±2460.76	234.37±211.06
	Nklc	Liver	6h	224.65±135.28	168.99±183.16
			12h	102.50±135.46	1.94±2.18
			24h	135.46±112.59	5.57±3.58
			48h	251.17±145.51	9.95±4.84
		Kidney	6h	138.97±7.19	283.95±161.04
			12h	46.25±120.68	0.02±0.02
			24h	120.68±29.08	40.85±6.03
			48h	109.90±73.97	104.72±29.51
	Nkld	Liver	6h	36.45±16.05	32.28±10.54
			12h	64.50±19.59	1.22±0.88
			24h	12.72±9.43	6.37±4.04
			48h	118.62±100.25	612.19±176.53
		Kidney	6h	75.87±50.28	155.22±80.33
			12h	48.84±19.30	0.85±0.31
			24h	106.92±32.30 (0.0347)	6.90±1.90
			48h	117.40±80.89	52.76±20.32
