# Supplementary material for: Rapid Natural Killer Cell Gene Responses, Generated by TLR Ligand-Induced Trained Immunity, Provide Protection to Bacterial Infection in rag1−/− Mutant Zebrafish (Danio rerio)
Source: Int J Mol Sci. 2025 Jan 23;26(3):962. doi: 10.3390/ijms26030962 (PMC11818001; doi:10.3390/ijms26030962)
Supplement: Supplementary file 1 [file ijms-26-00962-s001.zip › ijms-3360724-supplementary/Table S2 Flow Cytometry Statistical Analyses.pdf]

Supplemental Table S2. Flow cytometry statistical comparisons between 24 hour and 48 hours post beta glucan, R848 or RE33® exposure (primary) or between 24 and 48 hours post WT *E. ictaluri* exposure one month after beta glucan, R848 or RE33® exposure. Single asterisk * indicates p<0.05, double asterisk ** indicates p< 0.01 and triple asterisk *** indicates p<0.001.

Tissue/antibody	Treatment	Response	Summary	CI of diff	p value
Kidney NITR9	RE33®	Primary	***	-2.92 to 1.91	0.0006
Kidney NITR9	R848	Primary	ns	-7.18 to 5.72	1.0
Kidney NITR9	Beta glucan	Primary	**	-3.78 to -1.44	0.0083
Kidney NITR9	RE33®	Secondary	*	-38700 to -32.6	0.0497
Kidney NITR9	R848	Secondary	ns	-15050 to 23566	1.0
Kidney NITR9	Beta glucan	Secondary	ns	-15600 to 23017	1.0
Liver NITR9	RE33®	Primary	ns	-3.93 to 1.98	0.069
Liver NITR9	R848	Primary	**	-2.15 to -0.889	0.005
Liver NITR9	Beta glucan	Primary	*	-2.35 to -0.240	0.028
Liver NITR9	RE33®	Secondary	ns	-25700 to 27800	1.0
Liver NITR9	R848	Secondary	ns	-28800 to 24700	1.0
Liver NITR9	Beta glucan	Secondary	ns	-21300 to 32200	1.0
Kidney MPEG-1	RE33®	Primary	***	-1.47 to 0.664	0.0004
Kidney MPEG-1	R848	Primary	***	-1.47 to 0.665	0.0004
Kidney MPEG-1	Beta glucan	Primary	**	-1.80 to 0.376	0.0021
Kidney MPEG-1	RE33®	Secondary	ns	-281 to 231	1.0
Kidney MPEG-1	R848	Secondary	ns	-298 to 213	1.0
Kidney MPEG-1	Beta glucan	Secondary	ns	-191 to 320	1.0
Liver MPEG-1	RE33®	Primary	*	-1140 to -160	0.014
Liver MPEG-1	R848	Primary	ns	-720 to 257	0.510
Liver MPEG-1	Beta glucan	Primary	*	-991 to -14.0	0.045
Liver MPEG-1	RE33®	Secondary	*	0.019 to 1.93	0.047
Liver MPEG-1	R848	Secondary	ns	-1.37 to 1.53	1.0
Liver MPEG-1	Beta glucan	Secondary	*	0.130 to 1.38	1.0
