# Supplementary figures and images for: Rapid Natural Killer Cell Gene Responses, Generated by TLR Ligand-Induced Trained Immunity, Provide Protection to Bacterial Infection in rag1−/− Mutant Zebrafish (Danio rerio)
Source: Int J Mol Sci. 2025 Jan 23;26(3):962. doi: 10.3390/ijms26030962 (PMC11818001; doi:10.3390/ijms26030962)

**A. Kidney - *Ifny***

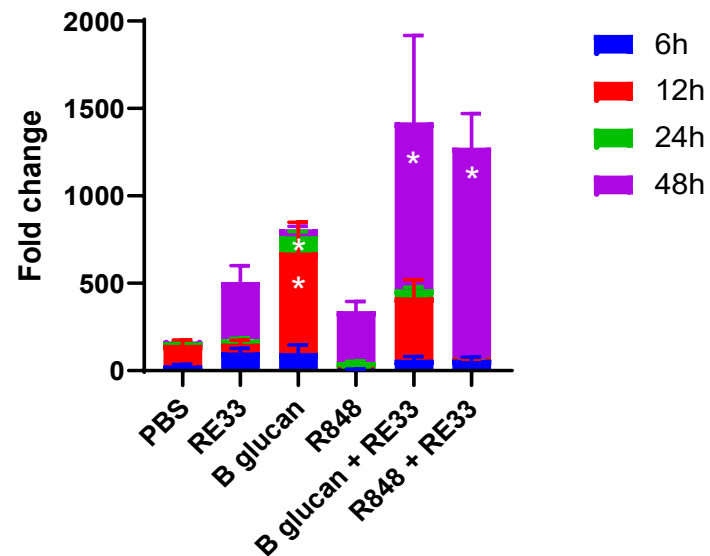

**B. Kidney - *Nitr9***

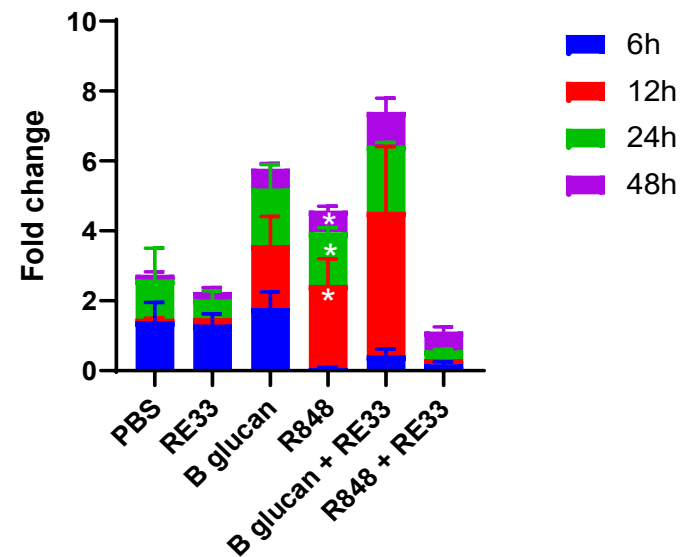

**C. Kidney - *Tbet***

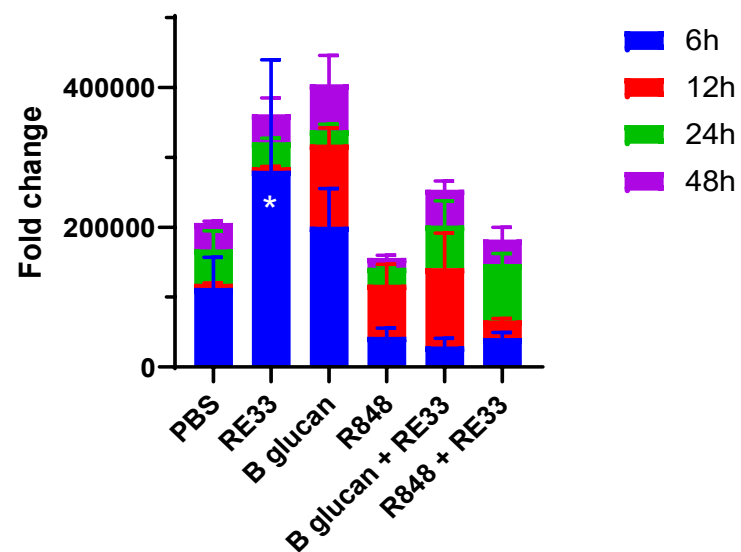

Supplement: Supplementary file 1 [file ijms-26-00962-s001.zip › ijms-3360724-supplementary/rapid gene response Supp Fig 1 qPCR kidney ifng, nitr9, tbet.pdf]

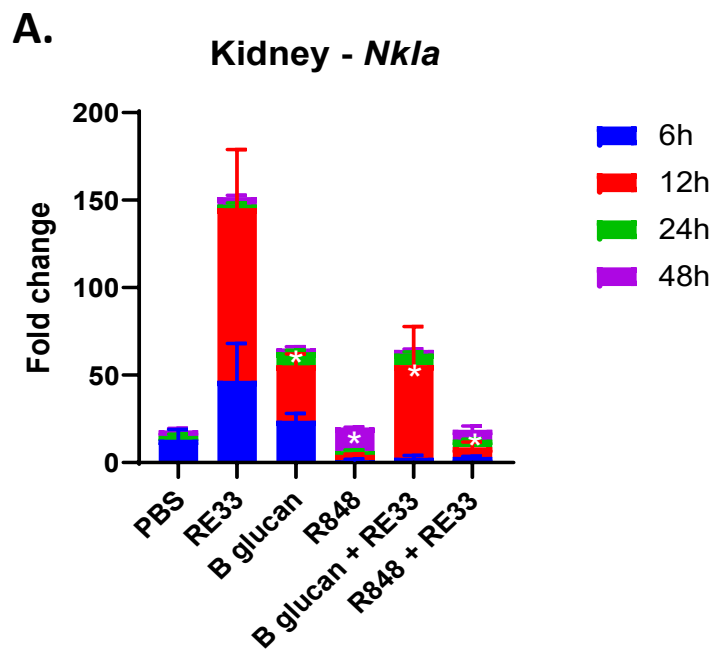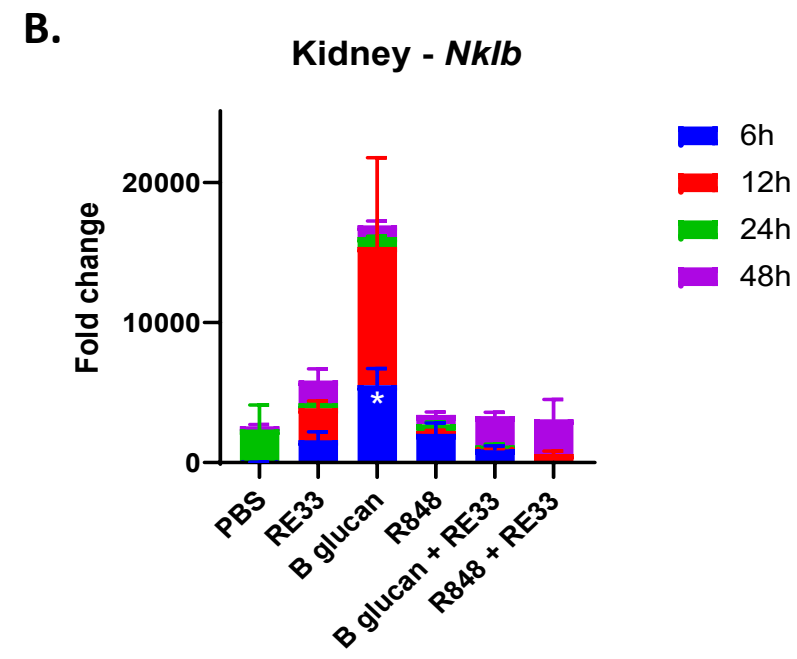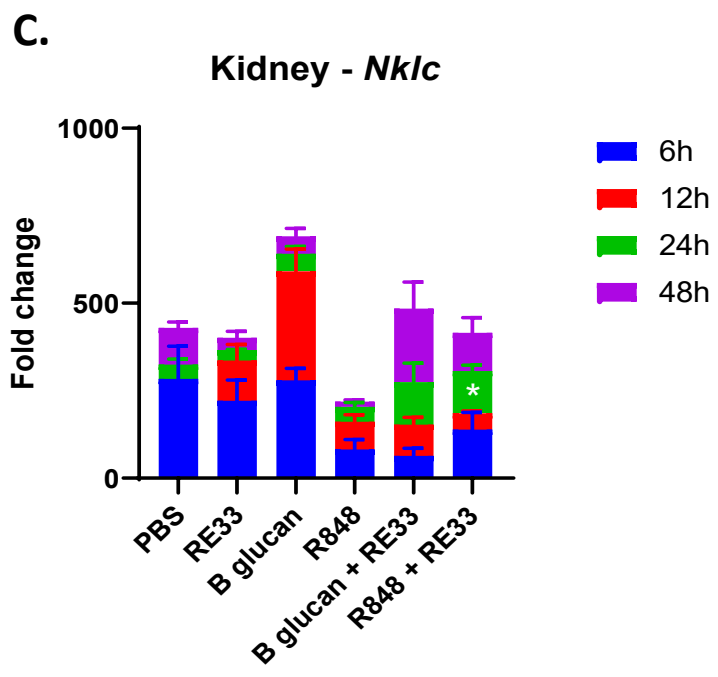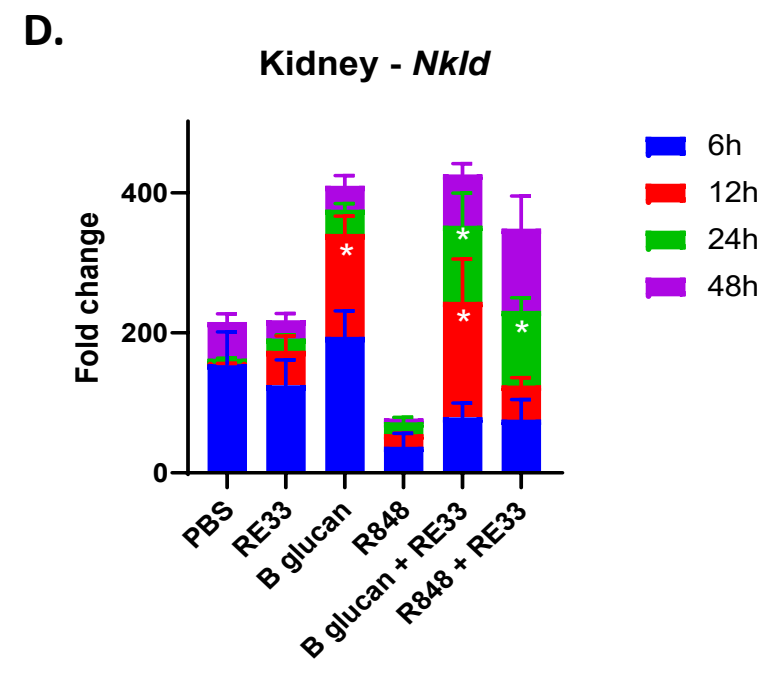

Supplement: Supplementary file 1 [file ijms-26-00962-s001.zip › ijms-3360724-supplementary/rapid gene response Supp Fig 2 qPCR kidney nk lysins.pdf]

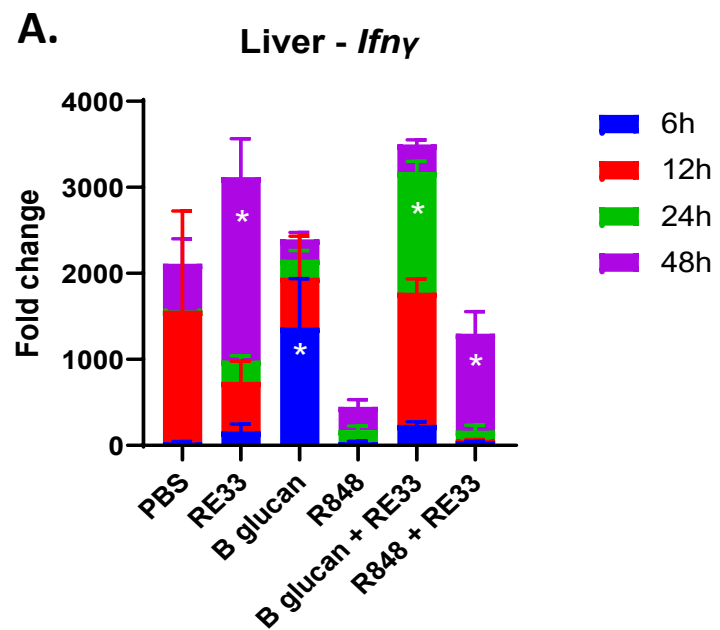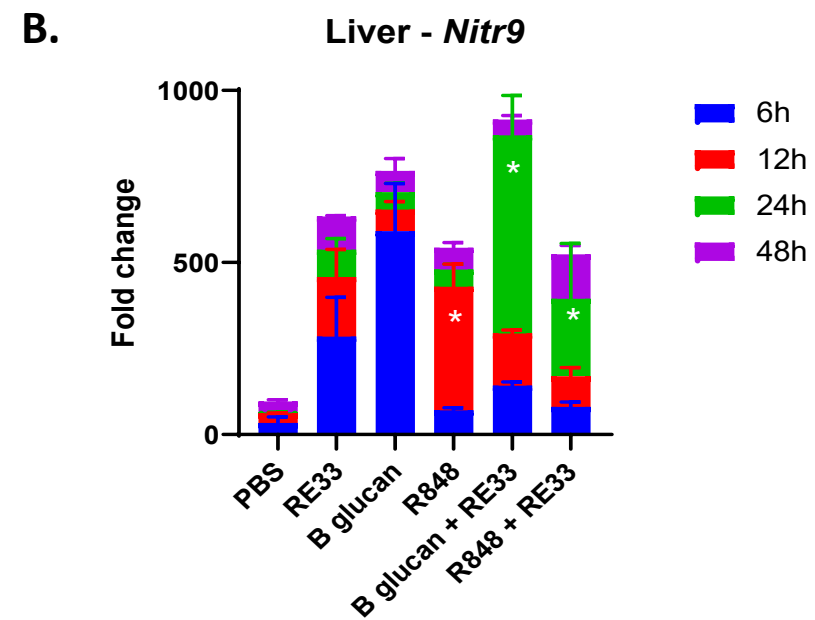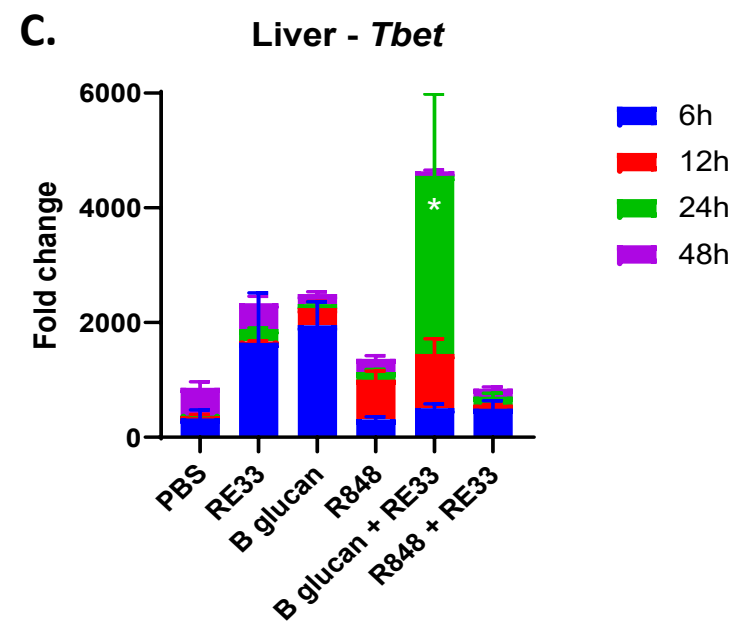

Supplement: Supplementary file 1 [file ijms-26-00962-s001.zip › ijms-3360724-supplementary/rapid gene response Supp Fig 3 qPCR liver ifng, nitr9, tbet.pdf]

**A.** Liver - *Nk1a*

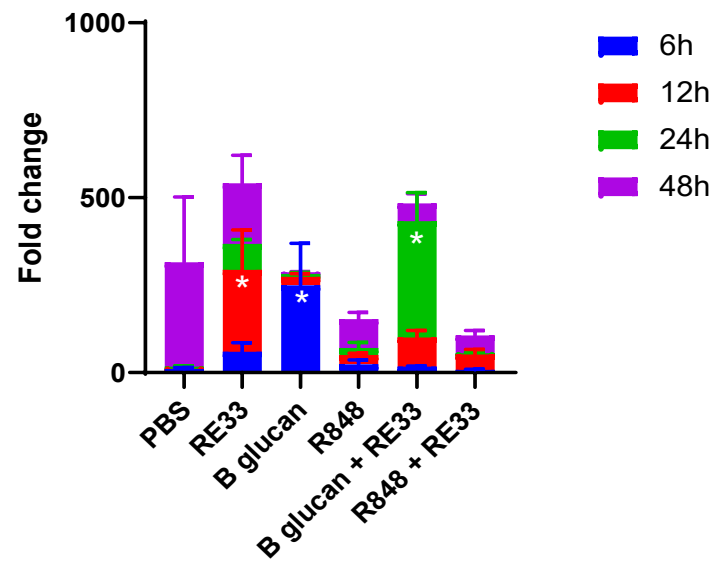

**B.** Liver - *Nk1b*

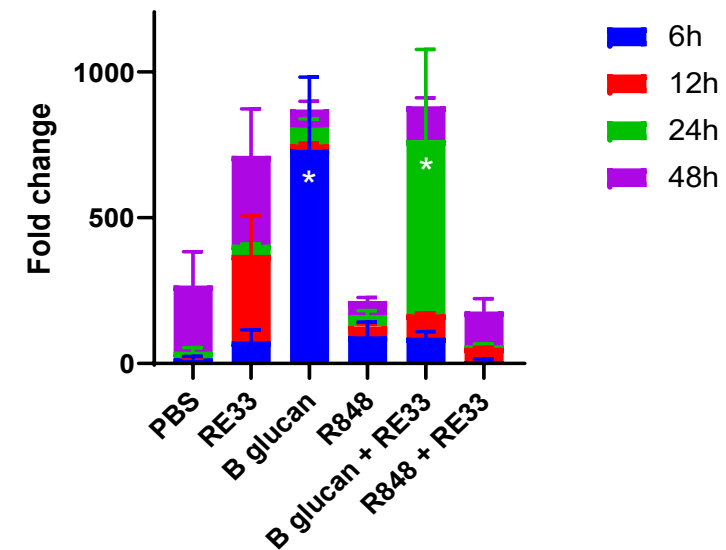

**C.** Liver - *Nk1c*

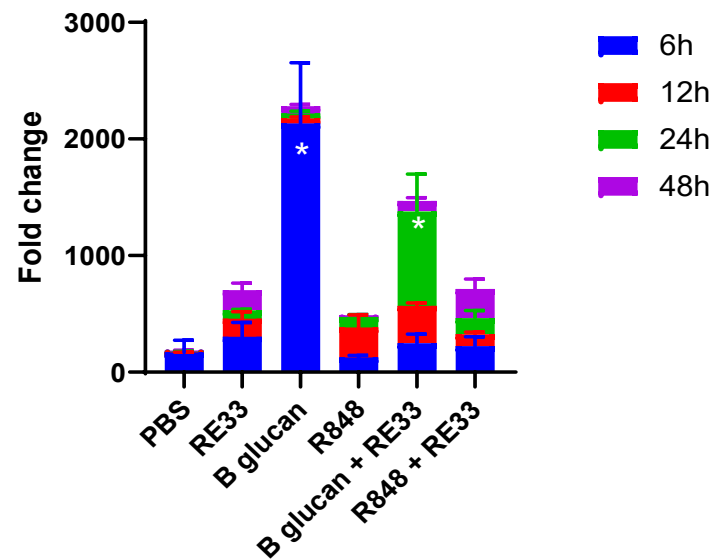

**D.** Liver - *Nk1d*

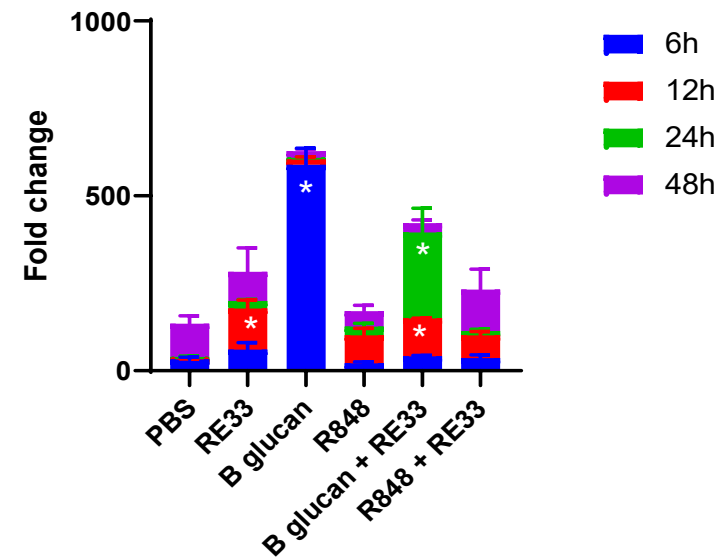

Supplement: Supplementary file 1 [file ijms-26-00962-s001.zip › ijms-3360724-supplementary/rapid gene response Supp Fig 4 qPCR liver nk lysins.pdf]

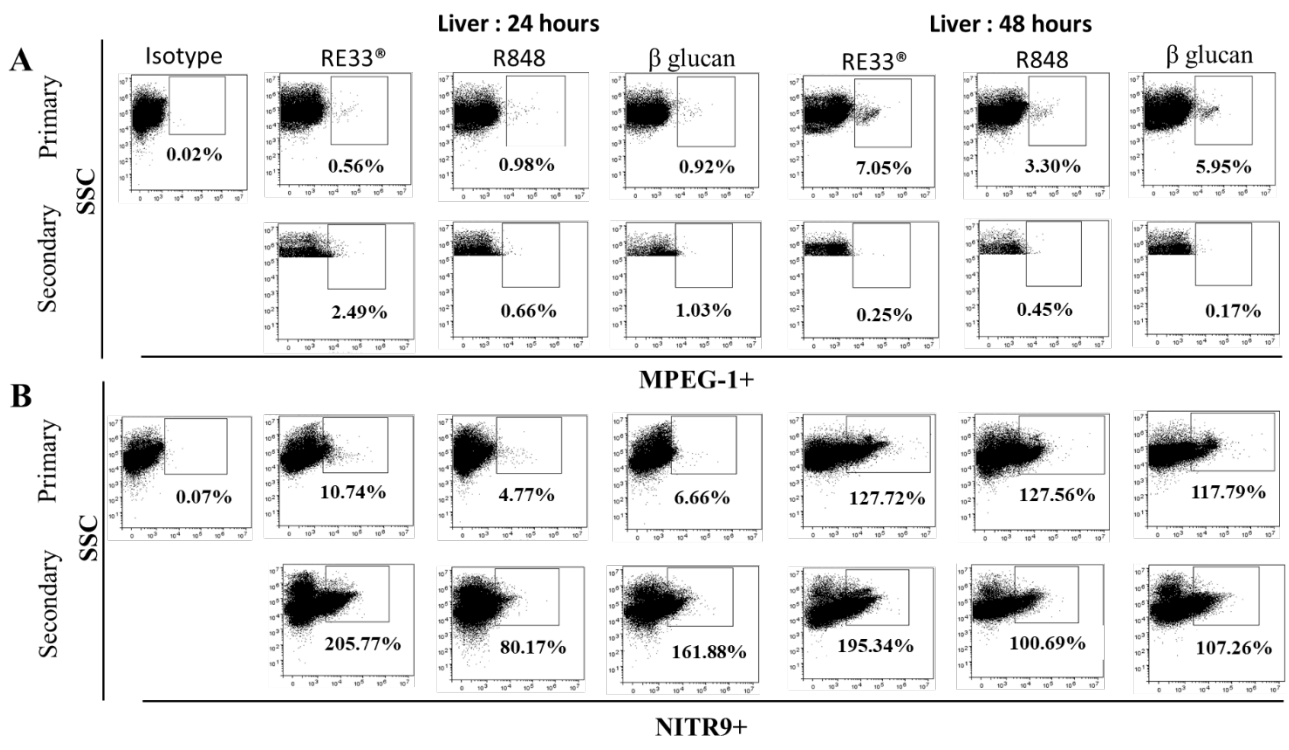

Supplement: Supplementary file 1 [file ijms-26-00962-s001.zip › ijms-3360724-supplementary/rapid gene response Supp Fig 8 liver FC.pdf]
